# Synchronous termination of replication of the two chromosomes is an evolutionary selected feature in Vibrionaceae

**DOI:** 10.1371/journal.pgen.1007251

**Published:** 2018-03-05

**Authors:** Franziska S. Kemter, Sonja J. Messerschmidt, Nadine Schallopp, Patrick Sobetzko, Elke Lang, Boyke Bunk, Cathrin Spröer, Jennifer K. Teschler, Fitnat H. Yildiz, Jörg Overmann, Torsten Waldminghaus

**Affiliations:** 1 LOEWE Center for Synthetic Microbiology–SYNMIKRO, Philipps-Universität Marburg, Marburg, Germany; 2 Leibniz Institute DSMZ-German Collection of Microorganisms and Cell Cultures, Braunschweig, Germany; 3 Department of Microbiology and Environmental Toxicology, University of California, Santa Cruz, Santa Cruz, United States of America; 4 German Centre of Infection Research (DZIF), Partner Site Hannover–Braunschweig, Braunschweig, Germany; Indiana University, UNITED STATES

## Abstract

*Vibrio cholerae*, the causative agent of the cholera disease, is commonly used as a model organism for the study of bacteria with multipartite genomes. Its two chromosomes of different sizes initiate their DNA replication at distinct time points in the cell cycle and terminate in synchrony. In this study, the time-delayed start of Chr2 was verified in a synchronized cell population. This replication pattern suggests two possible regulation mechanisms for other *Vibrio* species with different sized secondary chromosomes: Either all Chr2 start DNA replication with a fixed delay after Chr1 initiation, or the timepoint at which Chr2 initiates varies such that termination of chromosomal replication occurs in synchrony. We investigated these two models and revealed that the two chromosomes of various Vibrionaceae species terminate in synchrony while Chr2-initiation timing relative to Chr1 is variable. Moreover, the sequence and function of the Chr2-triggering *crtS* site recently discovered in *V*. *cholerae* were found to be conserved, explaining the observed timing mechanism. Our results suggest that it is beneficial for bacterial cells with multiple chromosomes to synchronize their replication termination, potentially to optimize chromosome related processes as dimer resolution or segregation.

## Author summary

Most bacteria encode their genetic information on a single chromosome. The pathogenic bacterium *Vibrio cholerae* is an exception to this rule and carries two chromosomes of different sizes, each having one origin of replication. A very basic research question is how the replication of the two chromosomes is timed starting from their replication origins. If they start simultaneously, the smaller chromosome would finish replication earlier than the larger chromosome. Interestingly, the timing in *V*. *cholerae* is such that the smaller chromosome starts replication after a time delay, resulting in synchronous replication termination of the two chromosomes. Here we answer the question whether it is the termination synchrony which is under evolutionary pressure, or whether a certain duration of the delay between the two chromosomes to start replication is of biological importance. To this end, we analyzed replication in different species of the Vibrionaceae phylogenetic group with differently sized chromosome pairs. Our results indicate that a synchronous termination of the two chromosomes in this group of bacterial species is under evolutionary selection, suggesting it to be potentially important for the process of cell division.

## Introduction

The diversity of regulatory systems of DNA replication has been studied in multiple bacteria [1–4]. An especially interesting group of bacteria with regard to DNA replication are those with multiple chromosomes. While a single chromosome is the norm in bacteria, about 10% of species in a diverse set of phyla carry more than one chromosome [5]. The best studied system in this respect is that of *V*. *cholerae*, the causative agent of the cholera disease [6, 7]. The genome of strain O1 El Tor N16961 is divided into two chromosomes of about 3 (Chr1) and 1 Mbp (Chr2) respectively [8]. Chr1 carries most of the essential genes [8, 9]. Replication at the origin of Chr1 (*ori1*) is initiated by the initiator protein DnaA, as is the case in almost all known bacteria [10]. Chr2 encodes its own initiator, RctB.[8, 11]. Notably, no RctB-like proteins have yet been found outside the phylogenetic group of Vibrionales. The structure of its central two domains (of four in total) resembles that of several plasmid replication initiators [12, 13]. RctB binds to a set of so-called iterons within *ori2* to initiate replication [14, 15], which contain the sequence GATC, methylated at the adenine by the Dam methyltransferase [16]. Binding of RctB was shown to be specific for fully-methylated GATCs, which in conclusion renders Dam essential in *V*. *cholerae*, unlike in *E*. *coli* [17–19]. RctB also binds to another type of sequence, the so-called 39-mers, which are also located at *ori2* [20]. However, the binding of RctB to the 39-mers does not activate replication as does its binding to the iterons; on the contrary, this suppresses initiation [20]. The balance between the activating and repressing action of RctB, in conjunction with a handcuffing mechanism, is thought to generate tight control of Chr2 replication in a cell-cycle-dependent manner [20, 21]. It was found that the two chromosomes start replication with a time delay in between [22–25]. In the search for regulatory mechanisms of communication between the two chromosomes, it was found that Chr1 was insensitive to the blockage of Chr2 replication [26]: it was shown that Chr1 controls replication of Chr2 through a short sequence about 800 kbp downstream from *ori1* [27]. This site was later named *crtS*, for ‘Chr2 replication triggering site’ [25]. Replication of *crtS* triggers the replication of Chr2, which is initiated after a short delay [25]. Moving the *crtS* site to other positions on Chr1 led to a corresponding shift in Chr2 initiation. The mechanism underlying the triggering effect of *crtS* is not yet fully understood but might involve physical contacts that were observed to occur between *crtS* and *ori2* [25].

Replication of Chr2 in *V*. *cholerae* starts after about two-thirds of Chr1 are replicated. This timing leads to termination of Chr2 replication at about the same time as that of Chr1. To better understand the mechanism underlying this phenomenon, we investigate here if it is the Chr2 replication starting after two-thirds of Chr1 is replicated which is important to the cell, or if the orchestrated termination of both chromosomes is the driving force of evolutionary selection. To this end, we tested whether the *V*. *cholerae* paradigm applies to other species of the Vibrionaceae and derive general rules of replication control.

## Results

### Delayed replication start of Chr2 of *V*. *cholerae* in a synchronized cell population

**Fig 1:**
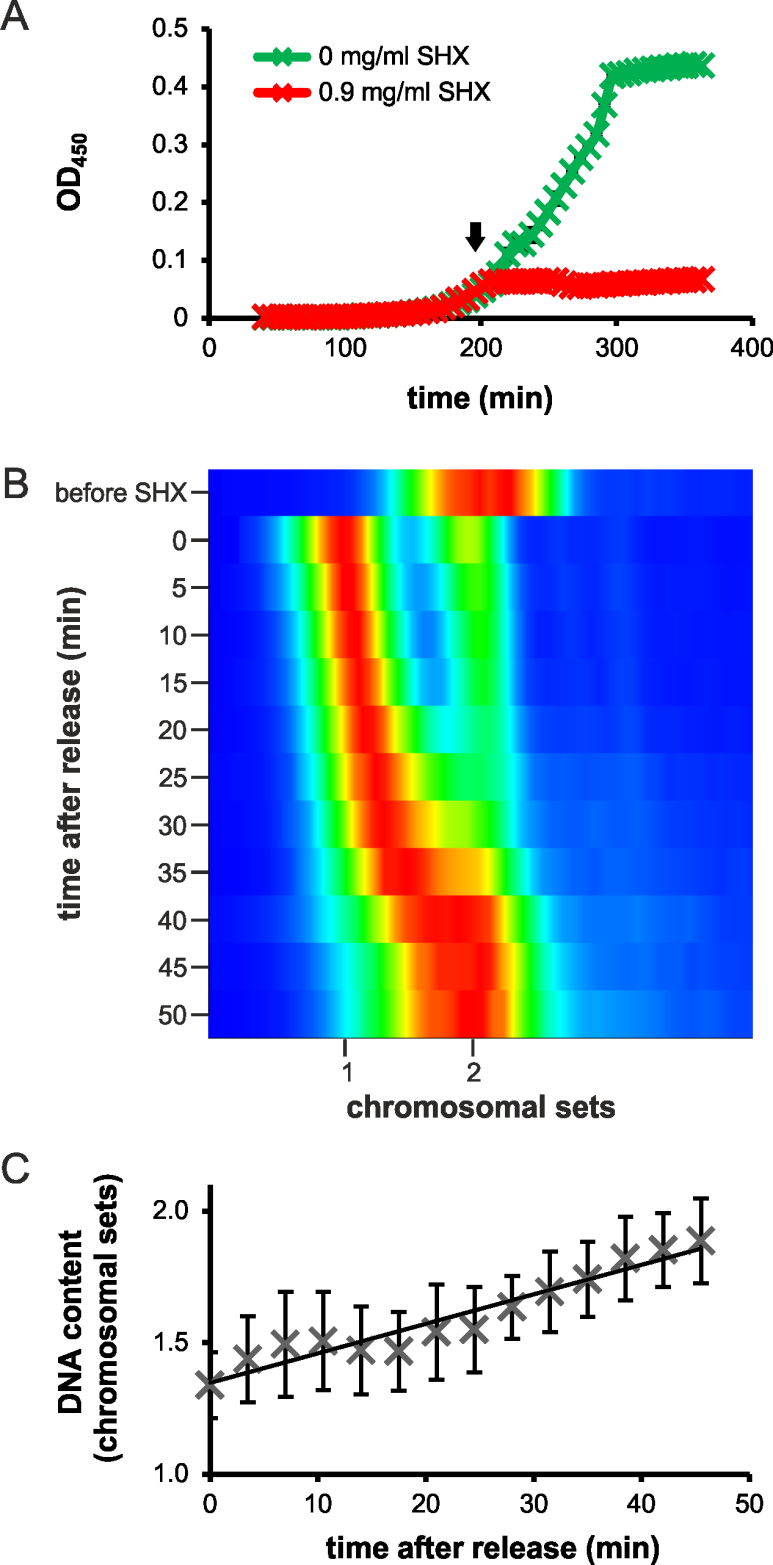
Synchronization of *Vibrio cholerae* by serine hydroxamate treatment. **(A)** Growth of *V*. *cholerae* A1552 without (green) and with (red) addition of SHX. Cells were grown in AB Glu CAA medium in a 96-well plate at 37°C. Addition of 0.9 mg/ml SHX is indicated by the black arrow. **(B)** Density maps of flow cytometry data of DNA-stained *V*. *cholerae* A1552. Exponentially grown cells in AB Glu CAA were treated with SHX for 150 min. After washing SHX off, samples were taken every five minutes as indicated. Cells were fixed with ethanol and stained with SYTOX Green. The flow cytometry data of the samples was aligned to the corresponding standard and converted into density maps. Red indicates a high density of cells, while blue—no cells. **(C)** Quantification of DNA content after release from SHX. Given numbers are mean values of three biological replicates as described in **(B)**. Error bars show standard deviations.

While early studies suggested a synchronous replication start of the two *V*. *cholerae* chromosomes, more recent studies support a time delay between Chr1 and Chr2 initiation [22, 23, 25, 28]. In synchronized *V*. *cholerae* cell cultures, such a time delay should lead to a situation with only Chr1 replicating in all cells short after initiation and later Chr2 replication. However, to date no synchronization method for *V*. *cholerae* has been available. Here we test if a synchronization method established for *Escherichia coli* can be used to synchronize *V*. *cholerae* populations [29]. The method is based on the induction of the stringent response as a cellular answer to nutrient limitation. In *E*. *coli*, addition of serine hydroxamate (SHX) blocks re-initiation of DNA replication, while ongoing replication rounds are finished, leading to cells with fully replicated chromosomes. Transfer of the cells to SHX-free medium then leads to a synchronous re-start of DNA replication. The stringent response in *V*. *cholerae* can also be induced by SHX treatment [30]. Consequently, addition of 0.9 mg/ml SHX to an exponentially growing *V*. *cholerae* batch culture resulted in clear inhibition of growth (Fig 1A). Flow cytometry analysis of the cellular DNA content shows asynchronous replicating cells before SHX treatment and cells with either 1+1 or 2+2 fully replicated chromosomes (Chr1 and Chr2) after SHX treatment for 150 minutes (Fig 1B). After transfer to growth medium without SHX, the DNA content of the cells increases gradually as would be expected for a synchronously replicating population (Fig 1B and 1C). To analyze replication of the two chromosomes individually, we performed marker frequency analyses using high density custom microarrays. We used the genome sequence of strain N16961 as a reference, which is very similar to the strain A1552 used here. A1552 (El Tor biotype, Inaba serotype) is a pathogenic strain of *V*. *cholerae* that was isolated from a traveler to Peru in 1992 (Strain DSM 106276 in the German Collection of Microorganisms and Cell Culture, DSMZ)[31]. However, during initial experiments we observed patterns indicating some sort of chromosomal rearrangements within the *V*. *cholerae* A1552 in comparison to strain N16961. For the latter, it was found that the strain used in most labs actually carries a chromosomal inversion between two operons encoding ribosomal RNAs ([25], Supporting S1A and S1B Fig. We used a set of PCRs to check potential additional inversions between ribosomal operons within the A1552 strain (Supporting S2 Fig). Results suggested a secondary inversion between rRNA operons A and C. To support this finding, we sequenced the A1552 genome with a combination of Illumina short read and Pacific Bioscience long read sequencing (GenBank Accession CP024867 and CP024868; see supporting S1 Text).

**Fig 2:**
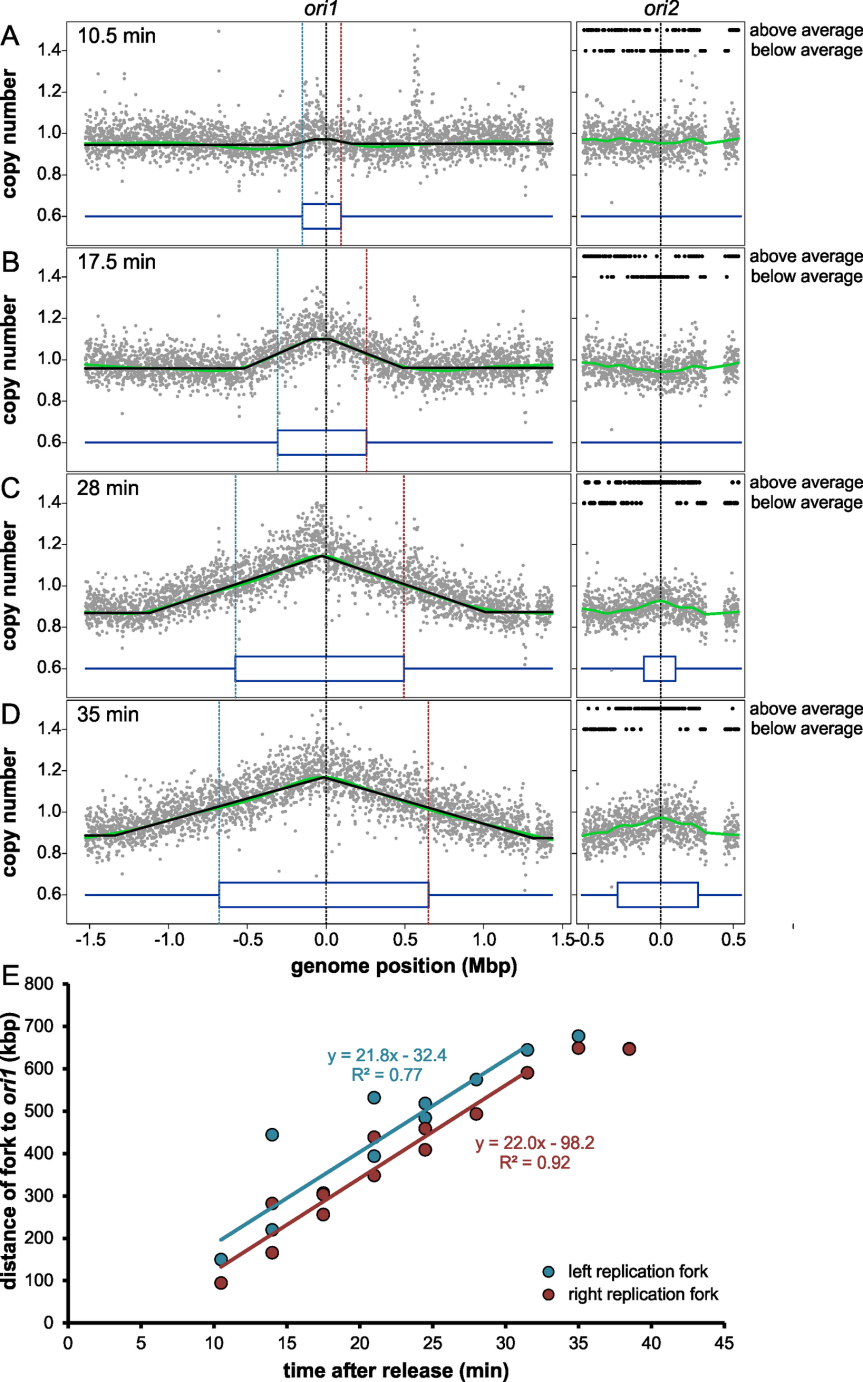
Chr1 of *Vibrio cholerae* starts its replication before Chr2 in a synchronized cell culture. (**A-D**) Profiles of genome-wide copy numbers at different time points after release from stringent response, based on comparative genomic hybridization. Grey dots represent mean values of 1kb windows. The genomic position is shown as the distance from *ori1* and *ori2*, respectively, indicated by dotted black lines. The green line corresponds to the Lowess regression (f = 0.2), and the black line on Chr1 to the fitted stepwise function for determination of average replication fork position. Mean positions of replication forks are shown by dotted blue (left) and red (right) lines. Black dots on Chr2 indicate if copy number values of 10 kb windows are above or below the average copy number of the corresponding time point as indicated. The large gap in Chr2 originates from exclusion of the superintegron due to a high frequency of non-unique sequences in this region [25]. Dark blue lines represent replicated (two lines) and non-replicated (one line) areas. For calculation details, see method section. (**E**) Progress of replication forks on both replichores after release from stringent response. Values of mean fork positions at different times after initiation (a total of 13 experiments) are plotted, with the left fork in blue and the right fork in red. Colored lines represent the respective regression which was calculated excluding the last two time points where replication reached the terminus.

Adjusting the genomic positions to the new genome sequence, we were able to follow the replication activity of the two *V*. *cholerae* chromosomes after release from the stringent response (Fig 2A–2D). Indeed, Chr1 initiated replication first as seen by a higher copy number of genomic loci near the replication origin 10.5 minutes after shifting to SHX-free medium. At later time points, this region of higher copy number increased gradually in size, indicating bi-directional replication towards the terminus region. Chr2 replication was not detected until 28 minutes after release from stringent response. Notably, its replication was more difficult to detect due to its smaller size and the large integron not providing reliable copy number values. A stepwise function was fitted to a total of 13 copy-number plots of Chr1 to determine average replication fork positions at different time points (Supporting S3 Fig)[32]. Interestingly, one replication fork runs about 60 kb ahead of the other on Chr1, similar to what was found for *E*. *coli* (Fig 2E). Based on the progression of the replication forks, we calculated a replication rate of 22 kbp/min or 360 bp/s for the replication in *V*. *cholerae* (Fig 2E).

### Position of *crtS* is linked to termination synchrony in Vibrionaceae

**Fig 3:**
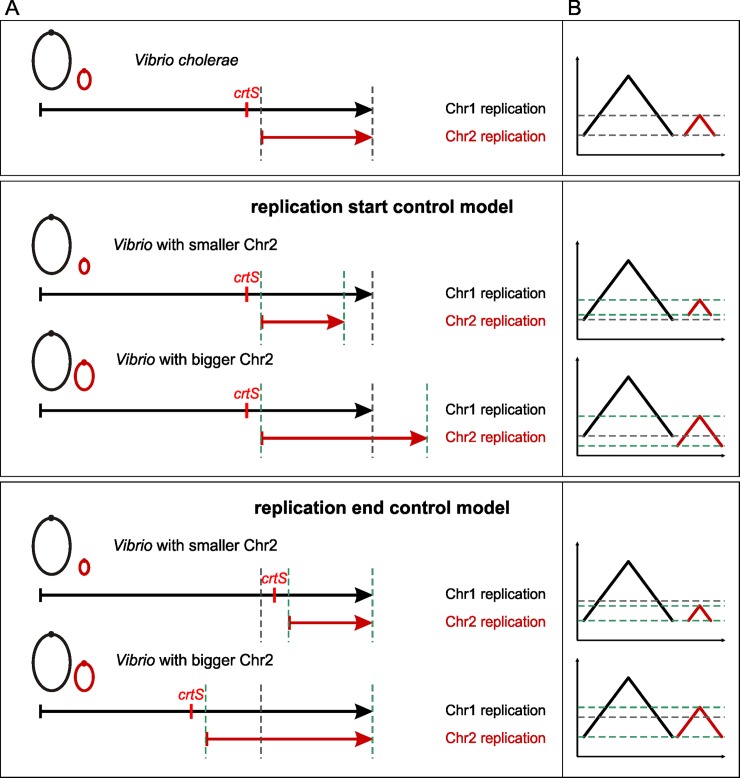
Replication start and end control models. (**A**) Scheme of replication patterns of *Vibrio cholerae*, replication start and end control model. Circles represent chromosomes, arrows the length and timing of DNA replication. Black stands for Chr1, red for Chr2. Grey dashed lines show start and end of DNA replication of *V*. *cholerae* Chr2. Green dashed lines indicate the corresponding expected start and end of DNA replication of Chr2. (**B**) Scheme of expected MFA plots of exponentially growing cells. Black lines represent regression lines for values of Chr1, red lines of Chr2. Dashed lines as in (**A**).

**Fig 4:**
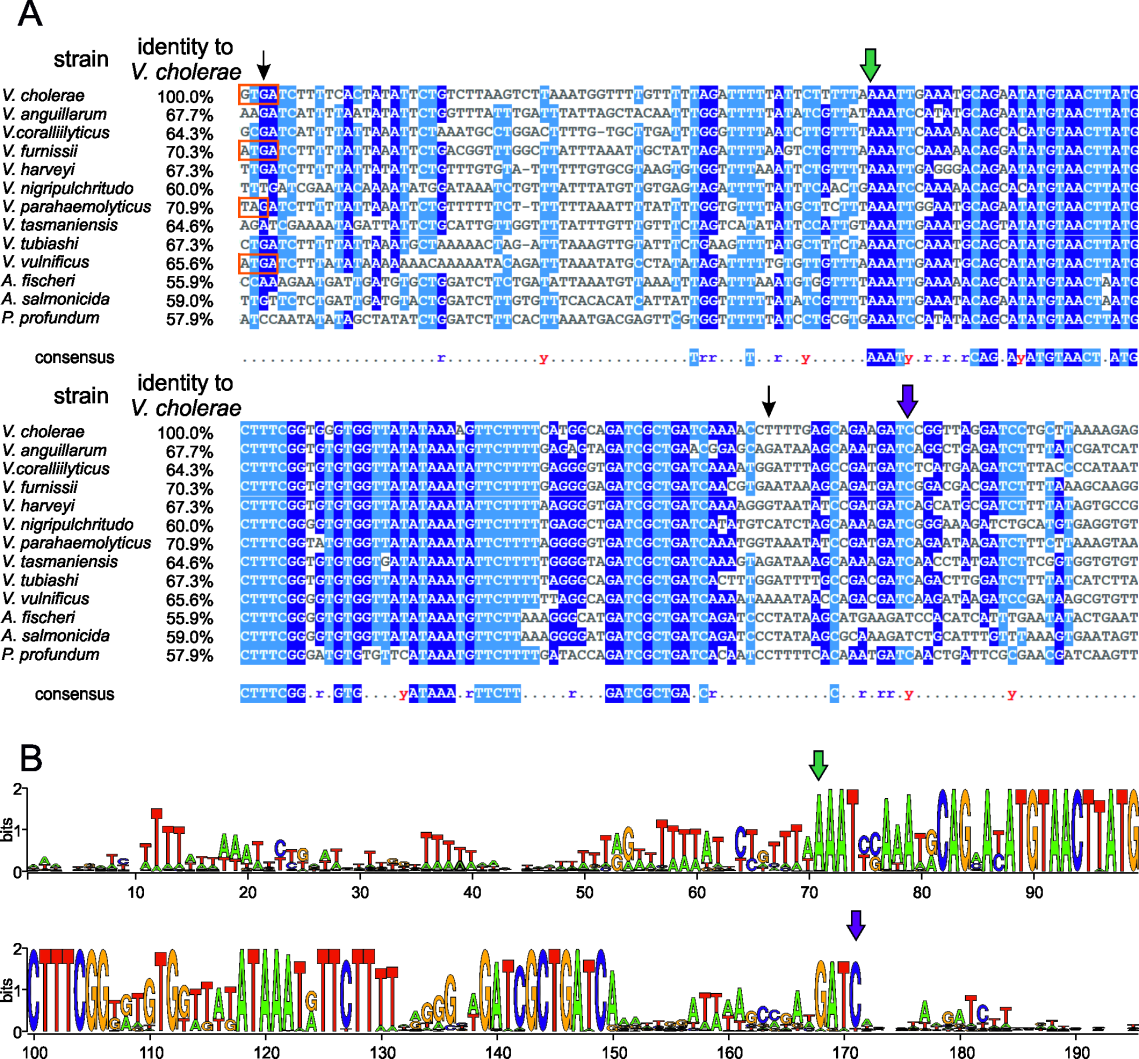
The *crtS* is conserved among Vibrionaceae. (**A**) Sequence alignment of *crtS* sites of 13 species within the Vibrionaceae, including *Vibrio*, *Aliivibrio* and *Photobacterium*. The alignment was done with Clustal Omega [67] and MView [68]. Blue highlights indicate sequence similarities, while orange rectangles the parts belonging to coding sequences of genes, black arrows start and end of the *crtS* as described [25]. Green and purple arrows are for orientation in comparing subfigure A and B. (**B**) WebLogo of *crtS* from 114 species of Vibrionaceae published on NCBI. The heights of letters corresponds to their conservation within the group of sequences [69].

**Fig 5:**
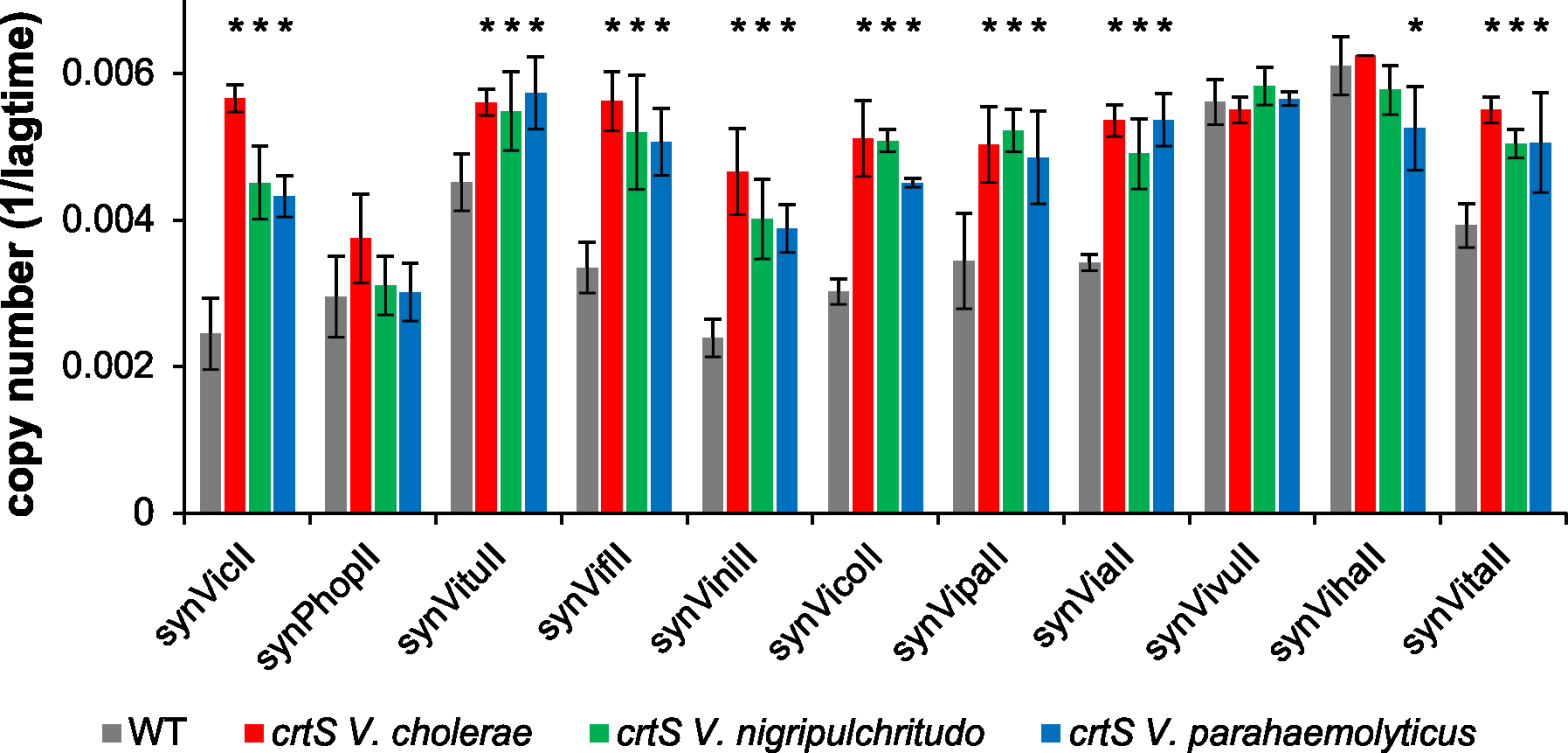
A *Vibrio crtS* increases the copy number of *ori2*-based mini-chromosomes in *E*. *coli*. Strains were grown in LB medium with 500 μg/ml ampicillin in a 96-well plate at 37°C. As strains with a lower replicon copy number need more time to start growth, the value 1 divided by the time to reach an OD_600_ ≥ 0.1 was used as measure for the replicon copy number. Grey bars represent the wildtype strain *E*. *coli* MG1655, with the corresponding mini-chromosome shown on the X-axis. Red, green and blue bars represent *E*. *coli* MG1655 with insertion of the *crtS* of *V*. *cholerae* (strain NZ139), *V*. *nigripulchritudo* (strain FSK103) or *V*. *parahaemolyticus* (strain FSK105), respectively. Values are the mean of three biological replicates with indicated standard deviation. A statistical significance in the difference between the WT and *crtS* strains is indicated by an asterisk (P≤0.05). Used mini-chromosomes are: synVicII-1.352 (*V*. *cholerae ori2*), synPhopII (*P*. *profundum ori2*), synVituII (*V*. *tubiashi ori2*), synVifII (*V*. *furnissii ori2*), synViniII (*V*. *nigripulchritudo ori2*), synVicoII (*V*. *coralliilyticus ori2*), synVipaII (*V*. *parahaemolyticus ori2*), synViaII (*V*. *anguillarum ori2*), synVivuII (*V*. *vulnificus ori2*), synVihaII (*V*. *harveyi ori2*), and synVitaII (*V*. *tasmaniensis ori2*). Growing the strains in standard concentrations of ampicillin did not show any difference between *wt* and *crtS* strains as expected (Supporting S4 Fig).

Clearly, the secondary chromosome in *V*. *cholerae* starts to replicate after about two-thirds of the primary chromosome are replicated, causing a synchronous termination of both chromosomes. Two different models could be derived from these observations for the DNA replication within the family of Vibrionaceae. First, it is of biological relevance to the cells to replicate two-thirds of the primary chromosome before starting replication of Chr2. Second, it is of biological relevance to the cells to terminate the two chromosomes at approximately the same time. Here we wanted to test both hypotheses to pave the way for a general understanding of the delay in initiation timing of DNA replication of Chr2 within the Vibrionaceae. The replication start control model, in which replication of two-thirds of Chr1 is important before Chr2 replication is initiated, implies that in *Vibrio* species with smaller secondary chromosomes than that of *V*. *cholerae*, replication of Chr2 ends before that of Chr1 (Fig 3A). In *Vibrio* species with larger secondary chromosomes, replication of Chr2 would terminate after Chr1. In contrast, the replication end control model would imply that replication of a small secondary chromosome starts later than that of a larger one (Fig 3A). To test both models we used a comparative genomics approach. Recently, replication of a sequence called *crtS* on Chr1 was found to trigger Chr2 initiation in *V*. *cholerae* [25, 27]. If such a site also appears in other *Vibrio* species, its position on Chr1 could be used as proxy for the time of Chr2 initiation. Based on the sequence of the *V*. *cholerae crtS* site, we searched the database for similar sequences occurring only once per genome in Vibrionaceae and generated a multiple sequence alignment (Fig 4A). The most conserved sequence parts were then used to find a set of 129 sequences and generate a corresponding sequence logo (Fig 4B). To test experimentally, if initiation at secondary replication origins in *Vibrio* species other than *Vibrio cholerae* is triggered by *crtS* sites, we analyzed the replication of mini-chromosomes, each driven by one of eleven secondary replication origins from different species of the *Vibrionaceae*. For a corresponding mini-chromosome based on *V*. *cholerae ori2*, it was shown that the copy number increases in an *E*. *coli* strain carrying a copy of the *crtS* site; this did not occur in a strain lacking *crtS* [27, 33]. As readout for the replicon copy number, we measured how well each strain tolerated increased amounts of antibiotic (Fig 5, see Method section for details, [34]). This method is based on the logic that an increased replicon copy number correlates with an increased copy number of the resistance gene, and so correlates with a higher antibiotic tolerance [35, 36]. A significant increase in copy number was observed for 8 out of 11 mini-chromosomes in an *E*. *coli* strain carrying the *V*. *cholerae crtS* site integrated into the chromosome in comparison to a strain without *crtS* (Fig 5, compare red and grey bars). Mini-chromosome copy number was similarly increased in strains carrying either the *V*. *nigripulchritudo* or the *V*. *parahaemolyticus crtS*. (Fig 5, green and blue bars). For two of the mini-chromosomes (synVivuII and synVihaII), the copy number appeared to be high already in the strain without *crtS* and one mini-chromosome (synPhopII) showed no *crtS*-dependent copy number increase (Fig 5). In summary, the data showed that replication origins of secondary chromosomes in *Vibrionaceae* are triggered by *crtS* sites in general, suggesting this mechanism to be conserved. The data also suggested that *crtS* sites do not function specifically on the *ori2* of their corresponding species, but appear to be interchangeable.

**Fig 6:**
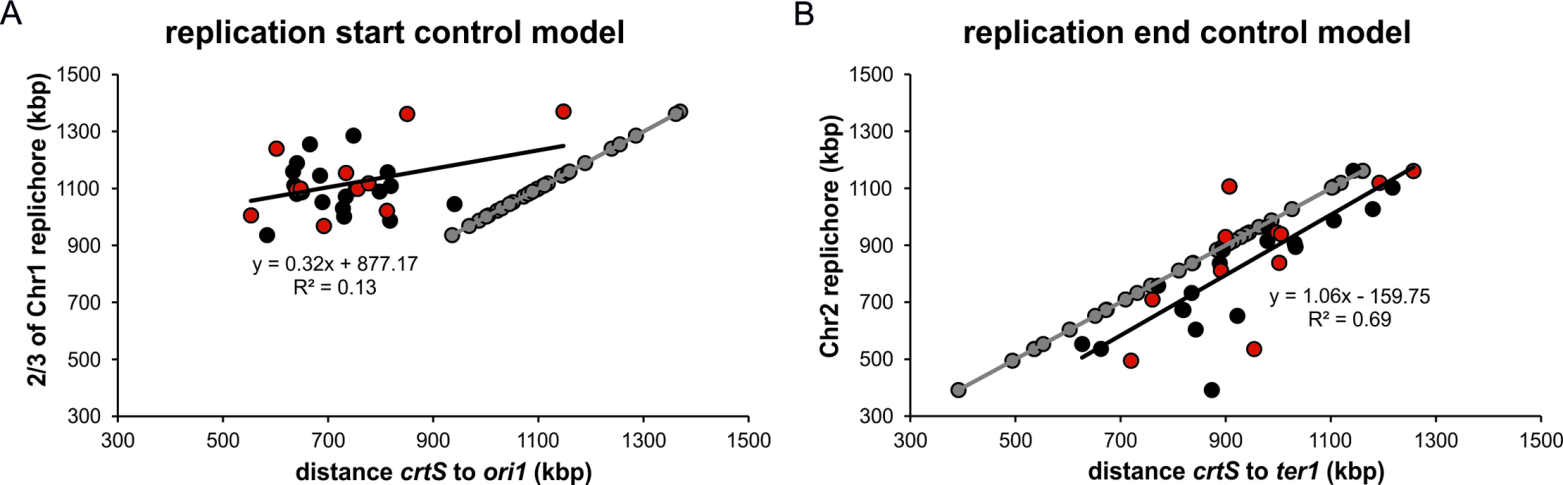
Comparative genomics support the replication end control model. Grey dots represent the expected values (full correlation of both parameters) of 29 fully sequenced Vibrionaceae (Supporting S1 Table), while black dots, the observed values. Red dots are the values of the strains further analyzed in Fig 7. Linear equations and R^2^ values are for regression of the observed values. A replichore is calculated as half of the corresponding chromosome, *ter1* is calculated as *ori1* + half of Chr1 (**A**) Examination of start control model. (**B**) Examination of end control model.

To test the replication start and end control models, the position of the *crtS* sites on the primary chromosomes of 29 fully sequenced Vibrionaceae species was determined, and the relative distance to *ori1* and *ter1* calculated (See method section for details). A correlation of the length of two-thirds of one Chr1 replichore to the distance of the *crtS* site to *ori1* would be expected if the start control model for replication in Vibrionaceae held true (Fig 6A, grey dots). However, the data from our comparative genomics approach showed no such correlation (Fig 6A, black and red dots). To test the replication end control model, the distance of the *crtS* site to *ter1* was plotted against the length of a Chr2 replichore (Fig 6B). Here, the values derived from comparative genomics resembled the theoretical data quite well, where the shift between the two respective regression lines correlate with the delay between *crtS* replication and *ori2* initiation observed in *V*. *cholerae* [25]. Our findings support the replication end control model to explain the replication timing of the two chromosomes in Vibrionaceae (Fig 3).

### Conservation of termination synchrony in Vibrionaceae

**Fig 7:**
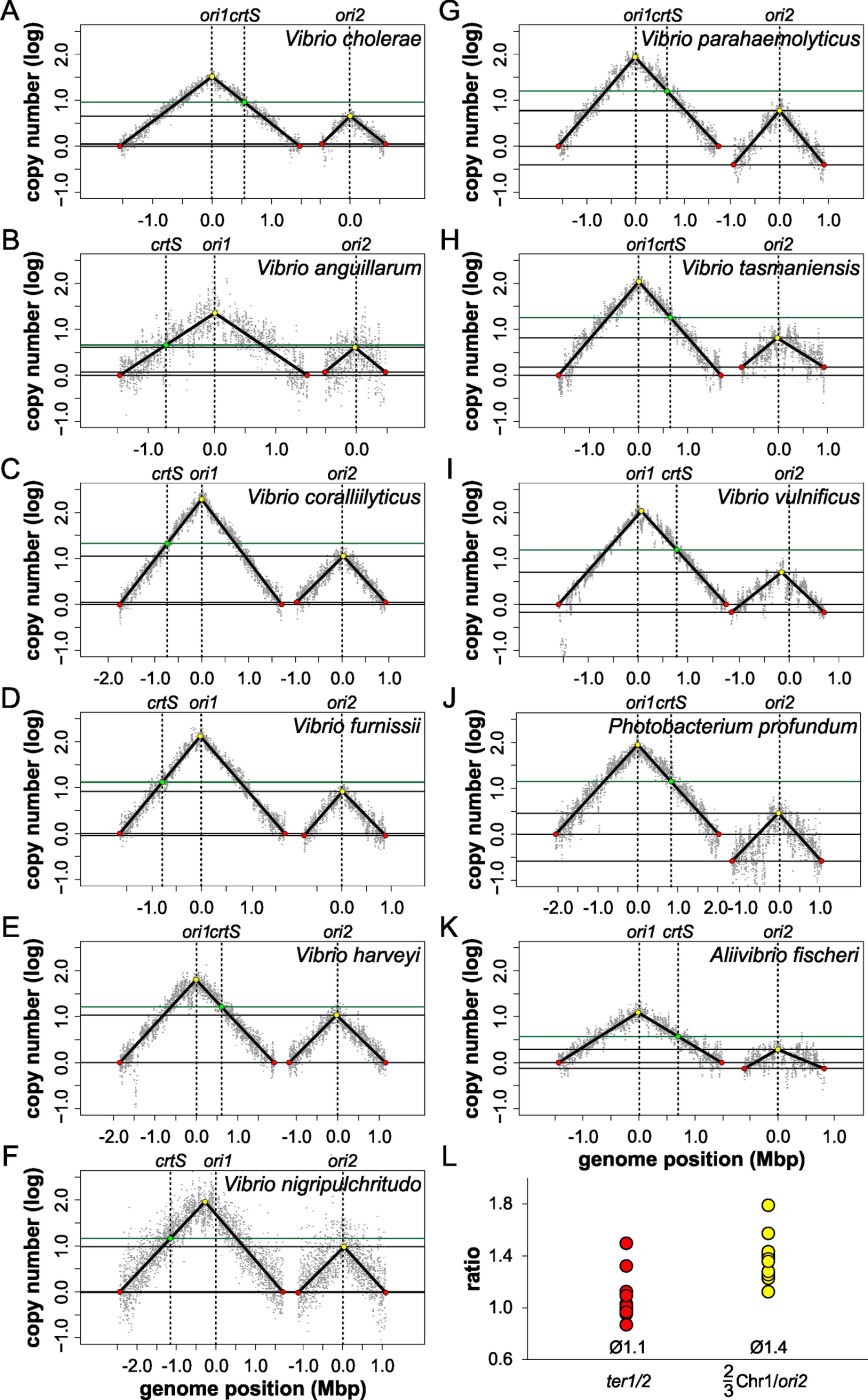
Conservation of termination synchrony in Vibrionaceae. (**A-K**) Profile of genome-wide copy numbers based on Illumina sequencing. Grey dots represent log numbers of reads as mean values for 5 kbp windows. The genome position is shown, indicated by vertical dotted black lines, as the distance from *ori1* and *ori2*, respectively. The *crtS* is also marked by a vertical dotted black line. The solid black lines represent the fitting of two regression lines. Maxima are highlighted in yellow, minima in red and the *crtS* in green. Horizontal lines show log copy numbers of *ter1*, *ter2*, *ori2* (all black) and *crtS* (green). Plots of biological replicates are shown in supporting S6 Fig. (**L**) Comparison of *ter1/2* copy number ratio (red dots) and the ratio of two-thirds of *ori1* copy number divided by *ori2* copy number (yellow dots). Mean values for both ratios are indicated.

To further test DNA replication in *Vibrionaceae*, we performed marker frequency analysis by next-generation sequencing of eleven different strains from the Vibrionaceae group. If the replication start control model holds true, one would expect copy numbers of *ter2* to be higher than *ter1* in *Vibrio* species where Chr2 is smaller than one-third of Chr1. In species with Chr2 bigger than one-third of the corresponding Chr1, the copy number of *ter2* should be below that of *ter1* (Fig 3B). If the replication end control model was applicable, one would expect the copy numbers of *ter1* and *ter2* to be equal in all *Vibrionaceae*. Chromosomal DNA was isolated from exponentially growing cultures and from cells in stationary phase. The DNA samples were then analyzed by Illumina sequencing and copy numbers plotted according to chromosomal positions. Copy numbers of the two chromosomes were close to one in stationary phase and showed a flat distribution in most cases, as expected for non-replicating cells (Supporting S5 Fig, supporting S4 Table). In all analyzed cases of exponentially growing cells, the copy number plots formed typical triangular shapes, with the replication origin at the highest point and copy numbers declining towards the termini (Fig 7). Note that the data were not normalized to the copy numbers of stationary phase cells. Two lines were fitted to each of the chromosomes and their intersection assigned as the minimal and maximal copy number as described [37]. The position of the maxima corresponded well with the positions of *ori1* and *ori2* with about 39 kbp deviation on average (below 1% of genome size), supporting good data quality (Supporting S2 Table). Also, data correlated well in biological replicates (Supporting S6 Fig, supporting S2 and S3 Tables). The copy number of *ori2* was lower than the copy number of *ori1* in all strains, consistent with a conserved replication mode within the Vibrionaceae. In fact, the copy number of *ori2* was also lower than that of the region carrying the corresponding *crtS* site on Chr1 in all studied strains, suggesting that *crtS*-based triggering of Chr2 replication is a conserved mechanism. The genomic plots showed the copy numbers of *ter1* and *ter2* within individual strains to be very similar, although some variation occurred (Fig 7). To test the two proposed models of replication start versus replication end control, we plotted the *ter1/ter2* ratio for the analyzed strains (Fig 7L). Values were around one, with some variation supporting the replication end control model. Notably, we found no good correlation between *crtS* position, Chr2 size and how well the *ter1/ter2* ratio matches 1. The ratio of copy numbers between the Chr1 position two-thirds of replichore size from *ori1* and the *ori2* copy number was higher, with a mean of 1.4. This indicates that replication in *Vibrionaceae* does not follow the replication start model (Fig 7L).

## Discussion

### Why is termination synchrony important?

In *V*. *cholerae*, the secondary chromosome initiates its replication after about two-thirds of the primary chromosome has been replicated. As a consequence, the two chromosomes terminate at approximately the same time. It has been shown that this replication pattern can be changed by moving the *crtS* sites to other positions, either further away or closer to the terminus [25]. Such engineered strains have no dramatic deficiencies in cell viability. Why has evolution shaped the replication timing to be as it is found in *V*. *cholerae* and other species of Vibrionaceae? We approached this question by asking if the selection pressure lies at the start or end of Chr2 replication timing (Fig 3). By analyzing replication rules in multiple *Vibrio* species, we show here that it is in fact the timing of the end of replication relative to Chr1 which is under selection, and not the start. In other words, the delay between the start of Chr1 and Chr2 replication respectively seems to be unimportant, but it appears to be more important that the two chromosomes terminate replication at approximately the same time. This begs the question of why synchronous termination of both chromosomes in Vibrionaceae is important. One reason could be the coordination of chromosome segregation and cell division. The chromosomal region opposite the replication origin is the part of the chromosome where dimer resolution occurs at the *dif* site [38]. In addition, chromosome segregation is coordinated with cell division through interactions of the Ter domain(s) with the divisome in *E*. *coli*, as well as in *V*. *cholerae* [39–41]. Interestingly, it was found that in engineered *V*. *cholerae* strains, in which Chr2 terminates long before Chr1, the two copies of *ter2* remain at the middle of the cell until cell division, and segregate approximately at the same time as *ter1*, like in wildtype cells [25]. Cohesion of *ter1* and *ter2* with their respective sister *ter* sequences near the division site thus seems to be important for segregation and the synchronized termination of the two chromosomes might facilitate this mechanism.

We could imagine an alternative explanation of why termination of the two chromosomes is conserved, which at present is more speculative. It could be that the replication pattern is the result of two opposing selection pressures. One driving force in *Vibrio* evolution could be the simultaneous replication of the two chromosomes. For cell cycle regulation and to limit the overall replication time to a minimum, it could be beneficial for the cell to not replicate one chromosome after the other. On the other hand, the secondary chromosome could be viewed as an invader which the cell needs to keep at bay as a second form of selection pressure. Indeed, Chr2 is thought to originate from a plasmid that the Vibrionaceae acquired early in evolution [20]. The cell might suppress Chr2 replication as far as it can to limit the danger of Chr2 taking over. Indeed, there is evidence that replication origins act as selfish genetic elements [42]. With overlapping replication cycles in fast growing cells, one could essentially imagine patterns in which Chr2 initiates replication before Chr1 with regards to the cell cycle. However, in different growth conditions, it is always Chr1 that initiates before Chr2 [22]. This finding might support a selective process active in *Vibrio* species to keep “Chr1 first”. In this context, it is also interesting that the initiator protein RctB actually has the capacity to mediate higher copy numbers of Chr2, since many different single amino acid changes lead to copy up of Chr2 numbers [14, 34, 43]. However, selection obviously works against these copy-up mutations in *Vibrios* occurring in nature. The selection pressure resulting from this Chr2 suppression would be to keep the Chr2 copy number as low as possible. The combination with the second selection pressure for simultaneous replication of the two chromosomes would finally result in termination synchrony. It is noteworthy that the termination synchrony appears to tolerate some deviation, as seen in the variation in copy number ratios of *ter1* to *ter2* (Fig 7). This observation suggests the evolutionary process leading to termination synchrony to be slow compared to chromosome rearrangements leading to changed distances between relevant genetic loci (*ori1*, *crtS*) within the system. One form of such chromosome rearrangement will be discussed in the next section. Besides the observed deviation in termination of the two chromosomes in *Vibrionaceae*, the delay between *crtS* replication and *ori2* initiation also appears to be variable (Fig 7). We did not find any correlation between this delay and other relevant parameters, such as *crtS* site position, Chr2 size, the distance of *ori1* to the *crtS* site, or between any combinations of these (Supporting S7 Fig). Factors influencing the delay duration remain to be discovered. One of the longest delays between *crtS* and *ori2* replication was observed for *Photobacterium profundum* (Fig 7J). Interestingly, the respective *ori2* mini-chromosome was not triggered by any of the three *crtS* sites tested in *E*. *coli* (Fig 4). The reason remains to be discovered since we found no obvious deviation of the *Photobacterium crtS*-site sequence from the other sequences (Fig 4). We also observed no increase of copy numbers for the mini-chromosomes with *ori2* copies of *V*. *harveyi* and *V*. *vulnificus* (Fig 5). However, here the copy numbers of mini-chromosomes were high already in strains without *crtS*. Notably, the used assay is not able to detect an even further increase in copy number. The copy number of the secondary chromosomes in *V*. *harveyi* and *V*. *vulnificus* are not increased, suggesting that the observed copy-up phenomenon is mini-chromosome specific.

### Inversions in strain A1552

Most published studies on DNA replication in *V*. *cholerae* have used the O1 El Tor strain N16961. This is the strain for which the first *V*. *cholerae* genomic sequence was published [8]. However, this strain was later found to not be transformable via natural competence due to a frameshift mutation in the regulator *hapR* [44, 45]. In contrast, the closely related O1 El Tor strain A1552 encodes a fully functional system of natural competence [44]. We thus decided to use strain A1552 for our experiments, expecting results directly comparable to studies using strain N16961, as we have frequently used the published N16961 genome sequence for primer design for A1552 sequences and never experienced any deviation. Our probe design for DNA microarrays was therefore also based on the N16961 genomic sequence. However, the initial plots showed non-continuous slopes from origin to terminus, indicating some sort of chromosomal rearrangement [46]. Interestingly, an inversion around *ori1* was also found recently in strain N1691, in contrast to the published genome sequence (Supporting S1B Fig and [25]). This inversion at ribosomal operons B-H was also detected in strain A1552. Additionally, a second inversion was found at rRNA operons A-C by diagnostic PCR and whole genome sequencing, including long reads to cover the large rRNA encoding operons. These operons were indeed the point at which the inversions occurred. This is certainly due to the high similarity of rRNA operons to each other presenting a good target for homologous recombination as mechanism of inversion. Such inversions at homologous sequences such as rRNA operons or transposons have been observed when comparing genome arrangements of related strains or during long term evolution experiments [47, 48]. A reconstruction of *Yersinia* evolution by comparative genomics suggests that as much as 79 inversions have happened to shape the genome arrangement seen today [49]. Inversions such as those found here in *V*. *cholerae* might thus happen quite frequently. This is certainly interesting in the context of chromosomal macrodomains that often rely on biased distributions of DNA motifs in the origin-to-terminus orientation [50–52]. However, it is also relevant for the *crtS*-based regulation of Chr2 initiation, in which chromosomal distances between *crtS* and origin or terminus certainly matter [25], as also seen in our study. In fact, the distance between the *crtS* site and *ori1* is changed by 142 kbp in the strain A1552 analyzed here compared to the N16961 strain analyzed before. This difference correlates well with an earlier termination of Chr2 relative to Chr1 in strain A1552 in comparison to N16961 (Fig 7, [25]). We cannot exclude that *crtS*-site positions in the different *Vibrio* species we analyzed are also re-localized slightly as, in some cases, we analyzed sub-strains not exactly resembling the strain with an available genome sequence. However, the analyses of many *Vibrios* instead of only individual strains should even out such uncertainties.

### Synchronization of *V*. *cholerae*

Bacterial populations in batch cultures are mixtures of cells in all different cell cycle stages, ranging from newly-born cells to large cells shortly before division. Such cultures can be used in many ways to study mechanisms of DNA replication and related processes. However, often a synchronized cell culture is desirable in studies investigating temporal resolution in DNA replication. In bacteria, different methodologies have been developed to generate synchronous populations. These include differential density centrifugation for *Caulobacter* and related bacteria [53], the baby machine [54], the baby cell column [55], and the blocking, and latter release, of DNA replication by the temperature shift of strains carrying temperature-sensitive protein mutants involved in initiation of DNA replication [56, 57]. The later approach was first developed in *E*. *coli* based on screens for mutant strains with temperature sensitive DNA replication mechanisms. Mutations appeared either in the initiator protein DnaA, or the DnaC protein responsible for loading the helicase DnaB. It was shown that amino-acid exchanges which rendered the *E*. *coli* DnaA temperature sensitive could help to rationally design a temperature sensitive DnaA in other bacteria [58]. Since *V*. *cholerae* and *E*. *coli* are relatively closely related and their DnaA proteins are highly similar, we mutated the *V*. *cholerae dnaA* according to the temperature sensitive *E*. *coli* DnaA. Notably, DnaC could not be used because *V*. *cholerae* lacks a homolog. The constructed *V*. *cholerae* DnaA showed temperature-sensitive activity in the heterologous *E*. *coli* system, but we were not able to exchange the natural *V*. *cholerae dnaA* with the mutated allele. As alternative way of synchronization, we tested the stringent-response-based method established by Ferullo and colleagues. [29]. Here, initiation of replication is blocked by addition of serine hydroxamate (SHX), which induces the stringent response. Transfer to SHX-free medium leads to synchronous initiation of DNA replication in *E*. *coli*. Ferullo and his collaborators suggested that this method should be transferable to other bacteria with a stringent response system such as that in *E*. *coli*. We demonstrated here that this assumption is true and predict that more, but not all, bacterial species could be synchronized using the stringent response. A negative example would be *Bacillus subtilis*, where the elongation of DNA replication, not the initiation, is blocked by inhibiting primase, an essential component of the replication machinery, during the stringent response [59]. Although we clearly showed the synchrony of the culture, including the linear increase of cellular DNA content as well as temporally-separated initiation of Chr1 and Chr2 replication, the synchronization could be further improved. In a perfectly synchronized culture, all cells would initiate DNA replication at the same point in time, which is hardly feasible with currently available synchronization methods. For example, the initiation of chromosomal replication in the widely applied DnaC-ts system in *E*. *coli* differs in the range of some minutes between cells [60, 61]. Furthermore, in the system established here, cells initiate replication after the first cells have started replication, as can be seen by the increasing copy number of *ori1* in comparison to *ter1* over time after release from the stringent response (Fig 2). One possibility to limit replication initiation to a narrower window could be to add SHX for a second time which should limit initiation to the time between release and re-addition of SHX.

### One replication fork on Chr1 replicated ahead of the other

The chromosomal replication origin in *E*. *coli* was found to be asymmetric, this being the root of an offset between the two replisomes [32]. The offset varied from strain to strain between 40 to 130 kbp. We observed a similar offset for the two replication forks on Chr1 in *V*. *cholerae* in synchronized cells (Fig 2E). Regarding the sequence of the replication origins, the replication fork that runs ahead is the same in *V*. *cholerae* and *E*. *coli*. It was suggested that the asymmetry of replication is caused by the asymmetry of the replication origin itself, where the initiator protein DnaA multimerizes on the right side to melt an AT-rich region on the left side [32]. Intuitively, one could imagine the replication to start more easily in the direction where no initiator complex sits in the way. In the context of Chr2 regulation, the observed offset is interesting because the exact time point of *crtS* site replication, and with it the time of Chr2 initiation, depends on *ori1* orientation. The frequent chromosomal inversions around *ori1* discussed above might consequently lead to frequent changes in Chr2 replication timing and might also explain the observed deviation from exact termination synchrony in other *Vibrios* (Fig 7).

## Materials and methods

### Bacterial strains, plasmids, oligonucleotides and culture conditions

All strains, plasmids and oligonucleotides used in this study are listed in supporting S5–S7 Tables. Unless indicated otherwise, cells were grown in LB medium, Marine broth, or AB medium supplemented with 25 μg/ml uridine, 10 μg/ml thiamine and 0.2% glucose with 0.5% casamino acids (AB Glu CAA) or 0.4% sodium-acetate (AB So-Ac) [62]. Antibiotic selection for *E*. *coli* was used at the following concentrations if not indicated otherwise: Ampicillin 100 μg/ml, kanamycin 35 μg/ml, spectinomycin 100 μg/ml. For growth curves, cells were grown in a 96-well plate at 37°C in a microplate reader (Victor X3 Multilabel Plate Reader, PerkinElmer). OD_450_ was measured every 6 min for 18 h.

### Construction of replicons and strains

*ori2*-based mini-chromosomes synVihaII and synVitaII were constructed as described in [34]: *ori2*s with *parAB* and *rctB* were amplified from gDNA of the respective strain. For synVihaII, the *ori2* region was amplified with primers 1515/1517 and 1410/1516 from gDNA of *V*. *harveyi*. For synVitaII, the *ori2* region was amplified with primers 1164/1557 from gDNA of *V*. *tasmaniensis*. Both *ori2* regions were assembled with AscI-digested synVicII-1.351 per Gibson assembly [63] and transformed in *E*. *coli* XL1Blue cells. For integration of *crtS* in *E*. *coli* MG1655, integration cassettes were constructed by MoClo assembly [64]. For pMA161, the *crtS* was amplified with primers 1474/1475 from gDNA of *V*. *nigripulchritudo* and for pMA892 with primers 1443/1444 from gDNA of *V*. *parahaemolyticus*. All PCR products were assembled in pMA349 by MoClo assembly as described in [65] and transformed into *E*. *coli* TOP10 cells. For pMA451, the backbone pMA327 was assembled with pICH50900, pMA709, pMA710, pMA431 and pMA161. For pMA454, the backbone pMA327 was assembled with pICH50900, pMA709, pMA710, pMA431 and pMA892. All assemblies were transformed in *E*. *coli* DH5α λpir cells. Integration cassettes were cut out with BsaI, integrated in *E*. *coli* AB330, transferred in *E*. *coli* MG1655 per P1-transduction and recombined to remove the resistance as described in [65]. *oriII*-based mini-chromosomes were added to wild type and *crtS* strains by conjugation or transformation.

### Synchronization of *Vibrio cholerae*

*V*. *cholerae* A1552 grown in AB Glu CAA was treated with 0.9 mg/ml serine hydroxamate (SHX) at an OD_450_ of around 0.15 (exponential phase). After an incubation of 150 min, the cells were harvested by centrifugation and re-suspended in fresh medium without SHX. Samples for flow cytometry and CGH were taken every 3.5 min if not indicated otherwise.

### Flow cytometry

Unless described otherwise, the cells were harvested and washed twice in TBS (0.1 M Tris-HCl pH 7.5, 0.75 M NaCl). They were fixed in 100 μl TBS and 1 ml 77% ethanol and stored at least overnight at 4°C. The samples were washed in 0.5 M sodium-citrate and treated with 5 ng/ml RNase A in 0.5 M sodium-citrate for 4 hr at 50°C. They were stained with 250 nM SYTOX Green Nucleic Acid Stain (Thermo Fisher Scientific) and analyzed on Fortessa Flow Cytometer (BD Biosciences). The SYTOX Green fluorescence was measured through a 530/30 nm bandpass filter. *V*. *cholerae* A1552 cells grown in AB So-Ac were fixed with ethanol and stained as described above. These cells served as the standard and were measured alternatingly with the samples. Data was processed with the software FlowJo (Treestar, Ashland, USA). For display as density maps, the sample data was aligned to the corresponding standard and converted into density maps by R.

### Comparative genomic hybridization (CGH)

CGH was performed as described [37]. For hybridizing, Agilent SurePrint G3 Custom CGH Microarrays, 8x60K (Design ID: 074887) were used. They were designed on *V*. *cholerae* N16961 (NC_002505.1 and NC_002506.1) with a probe length of 60 bp and a probe distance of 7 bp. Probes with multiple hybridization sites were excluded. As a reference, DNA from stationary *V*. *cholerae* A1552 grown in AB Glu CAA was used. Probe signal ratio values were merged in 1000 bp windows. A Lowess fitting was applied to the microarray data to get a locally weighted average (shown as green line in CGH plots). For Chr1, a stepwise function was fitted on the data. The stepwise function divides the plot into five parts: two flat parts at the edges (not yet replicated), one flat risen part in the middle (already replicated) and an increasing and decreasing part (replicating at that moment). The four points at the transition from one of these lines to the next were defined by chromosomal position (x1 to x4) and the heights (h1 to h4). These values were estimated based on plots of the raw data and then used for fitting in conjunction with the stepwise function using nls (nonlinear least-squares) of the R statistics software. Mean positions of the replication forks were calculated as the middle of the increasing (left fork) or decreasing (right fork) part. Progression and asymmetry of the forks was calculated in Excel. For Chr2, copy number values of 10.000 bp windows were compared to the mean copy number of the corresponding plot and displayed as above or below the mean.

### Multiple sequence alignment

*crtS* sequences of Vibrionaceae in Fig 4A were found with BLAST [66]. The alignment was done with ClustalOmega [67] and the layout with MView [68]. Additionally, *crtS* sites in 114 Vibrionaceae from NCBI were found with fuzznuc (http://www.hpa-bioinfotools.org.uk/pise/fuzznuc.html) by using the sequence CAGnATATGTAACTnATGCTTTCGG with a maximum of three mismatches. This search resulted in only one hit per genome. The consensus was visualized with WebLogo 2.8.2 [69].

For comparative genomics of Vibrionaceae, data of 29 fully sequenced and annotated strains from NCBI were used. Positions of *ori1* and *ori2* were either found at dOriC [70] or by assigning the intergenic region between *gidA/mioC* (*ori1*) or *rctB/parAB* (*ori2*). The genes were either found by annotation or with BLAST [66]. One half of each chromosome was defined as a replichore, and *ter1* was then calculated as the opposite position to *ori1* on Chr1 (*ori1* + 1/2 Chr1). The position of *crtS* was found using the consensus sequence with fuzznuc. Expected values of both parameters were calculated by using the same data on both X and Y axis (two-thirds Chr1 replichore and Chr2 replichore, respectively).

### Mini-chromosome copy number analysis

Analysis of mini-chromosome copy numbers was as described [34]. Cells were grown in LB medium containing either 100 or 500 μg/ml ampicillin at 37°C in 96-well plates in a microplate reader (infinite M200pro multimode microplate reader, Tecan). The 150 μl of main culture was inoculated 1:1000 and growth curves recorded for 15 hr. Statistical significance of differences between *wt* and *crtS* strains was calculated by a two sample t-test. For better visualization, 1 divided by the time needed to reach an OD of 0.1 was defined as a measure of the copy number.

### Sequencing

For Illumina sequencing of the Vibrionaceae, cells were grown in marine broth at either 28°C or 10°C (*Photobacterium profundum*) to either exponentially or stationary phase. Genomic DNA was prepared by incubating resuspended frozen cell pellets in 300 μl TE-buffer with 1.2% SDS and 4 mM EDTA for 5 min at 65°C. After adding 750 μl isopropanol, the precipitate was incubated in 500 μl TE with 50 μg RNase A for 90 min at 65°C and additional 15 min at 37°C with 50 μg proteinase K. DNA was isolated with phenol/chloroform. Final DNA was resuspended in deionized sterile water and quantified using a NanoDrop (ThermoFisher Scientific). Genomic DNA was sequenced by applying the Nextera XT library kit and a MiSeq v3 reagent kit with 150 cycles on an Illumina MiSeq (Illumina, USA).

For PacBio sequencing, *V*. *cholerae* A1552 [31] was grown in AB Glu CAA at 37°C to stationary phase. Genomic DNA was prepared as above. Final DNA was resuspended in TE-buffer and quantified using a NanoDrop (ThermoFisher Scientific) and a Qubit Fluorometer (Life Technologies). The SMRTbell template library was prepared according to the instructions from PacificBiosciences, Menlo Park, CA, USA, following the Procedure & Checklist—20 kb Template Preparation Using BluePippin Size-Selection System. Briefly, for preparation of 15kb libraries ~8μg genomic DNA libraries was sheared using g-tubes from Covaris, Woburn, MA, USA according to the manufacturer´s instructions. DNA was end-repaired and ligated overnight to hairpin adapters applying components from the DNA/Polymerase Binding Kit P6 from Pacific Biosciences, Menlo Park, CA, USA. Reactions were carried out according to the manufacturer´s instructions. BluePippin Size-Selection was performed according to the manufacturer´s instructions with a size selection cutoff of 4 kb (Sage Science, Beverly, MA, USA). Conditions for annealing of sequencing primers and binding of polymerase to purified SMRTbell template were assessed with the Calculator in RS Remote, PacificBiosciences, Menlo Park, CA, USA. SMRT sequencing was carried out on the PacBio RSII (PacificBiosciences, Menlo Park, CA, USA) taking one 240-minutes movie.

### Genome assembly of *V*. *cholerae* A1552

Genome assembly was performed with the RS_HGAP_Assembly.3 protocol included in SMRT Portal version 2.3.0. Both chromosomal contigs were successfully assembled and trimmed, circularized, as well as adjusted to *dnaA* (Chr1) and *rctB* (Chr2) as the first gene.

Quality improvement of the PacBio HGAP assembly was performed by a mapping of all corresponding Illumina short reads using the Burrows-Wheeler Aligner (BWA) using bwa aln and bwa sampe [71]. Illumina reads were mapped onto the obtained chromosome and plasmid sequences with subsequent variant and consensus calling using Varscan2 [72] and GATK [73]. A final quality score of QV60 was attained. Automated genome annotation was carried out using Prokka [74]. The genome sequence was submitted to GenBank (Accession Number: CP024867; CP024868).

### Marker frequency analysis

In the first step, reads from the exponential and stationary phase were mapped on the respective *Vibrio* replicons using qalign from the QuasR R package. Subsequently, replicon-wide coverage was calculated by bedtools genomecov using the 5' ends of the reads. Single base coverage was smoothed by a 5 kbp sliding window averaging with a shift of 1 kbp. Windows with an internal standard deviation that exceeded three times the difference between the median and the third quartile of standard deviations of windows within 500 kbp were removed. These are windows, with an average coverage that does not properly reflect the coverage of individual bases. Furthermore, windows with an internal standard deviation below three times the difference between the median and the 1st quartile of standard deviations of windows within 500 kbp were removed. These are windows with many bases of low or mostly zero coverage indicating deviations of reads from the template sequence. The procedure removes unreliable window averages (data points) taking the noisiness of the data and regional specificities into account. Sequence bias was removed as follows: firstly, the coverage of exponential and stationary phase samples was normalized to the total amount of mapped reads to remove the bias of total read counts in the samples. Then, ratios of exponential and stationary phase coverage were determined. Ratios were subsequently corrected for a systematic sequence-dependent local bias [25], using the second exponential phase sample.

## Supporting information

S1 Fig Differences in genomes of commonly used *Vibrio cholerae* created by inversions. Circles represent Chr1, with green arrows indicating position and orientation of rRNA operons, and rectangles the position of *ori1*, *crtS* and *difI*. Colored lines show the distances between the rRNA operons, arrowheads are for orientation between the maps. Black arrows highlight the inversions. (**A**) Map of *V*. *cholerae* O1 El Tor N16961 Chr1 as described [8]. (**B**) Map of *V*. *cholerae* O1 El Tor N16961 Chr1 as described [25]. (**C**) Map of *V*. *cholerae* A1552 Chr1 as described here. (**D**) Size of the distances between the rRNA operons, based on NC_002505.1.
(TIF)

S2 Fig Verification of inversions in *V*. *cholerae* A1552 with colony PCR. (**A**) Map of *V*. *cholerae* N16961 *chrI*. Green lines represent rRNA operons, yellow, blue and red lines *ori1*, *crtS* and *difI*, respectively. Purple lines show the binding position of the used primers for diagnostic PCR on the forward (outer ring) and reverse strand (inner ring). Primer sequences are provided in supporting S7 Table. (**B**) Agarose gels of colony PCR on *V*. *cholerae* N16961 (strain “N”) and *V*. *cholerae* A1552 (strain “A”). Tested operons and primer combinations are indicated by green letters. All PCRs should give a product of approximately 6 kb, except GH, B_fw_-GH_rv_ and D in A1552, which should yield a product of 12 kb (see red asterisks). False 6-kb products in these cases are probably due to sequence similarity in the neighboring rRNA operons.
(TIF)

S3 Fig Marker frequency analysis of synchronized *V*. *cholerae* Chr1. (**A-I**) Profiles of additional replicates and time points of genome-wide copy numbers after release from stringent response. Derived data are summarized in Fig 2E.
(TIF)

S4 Fig Growth of *E*. *coli* with and without *crtS* and *ori2*-based mini-chromosomes in standard ampicillin concentrations. Strains were grown in LB medium with 100 μg/ml ampicillin in a 96-well plate at 37°C. Annotation is as in Fig 5.
(TIF)

S5 Fig Marker frequency analysis of different species of *Vibrionaceae* in stationary growth phase. (A-K) Profiles of genome wide copy numbers based on Illumina sequencing. Grey dots represent numbers of reads (normalized to a mean Chr1 copy number of 1). Black lines indicate the mean copy number of each chromosome.
(TIF)

S6 Fig Marker frequency analysis of different Vibrionaceae. (**A-K**) Profile of biological replicates of genome wide copy numbers shown in Fig 7 (similar annotation).
(TIF)

S7 Fig The delay between *crtS* replication and *ori2* initiation is larger than expected. The theoretic delay plotted against the real delay. Theoretic delay is the difference of one half of Chr2 and the distance between *crtS* and *ter1*. The real delay is the distance of *crtS* and the position on Chr1 with the same copy number as *ori2* according to the MFA data. Red dots are values from all analyzed strains in Fig 7. The black line has a slope of 1 and simulates perfect correlation.
(TIF)

S1 Table Strains used in comparative genomics. (PDF)

S2 Table MFA data of Fig 7. (PDF)

S3 Table MFA data for supporting S6 Fig. (PDF)

S4 Table MFA data of stationary phase. (PDF)

S5 Table Strains used in this study. (PDF)

S6 Table Replicons used in this study. (PDF)

S7 Table Oligonucleotides used in this study. (PDF)

S1 Text Sequencing of *V*. *cholerae* strain A1552. (PDF)
