# Supplementary figures and images for: Synchronous termination of replication of the two chromosomes is an evolutionary selected feature in Vibrionaceae
Source: PLoS Genet. 2018 Mar 5;14(3):e1007251. doi: 10.1371/journal.pgen.1007251 (PMC5854411; doi:10.1371/journal.pgen.1007251)

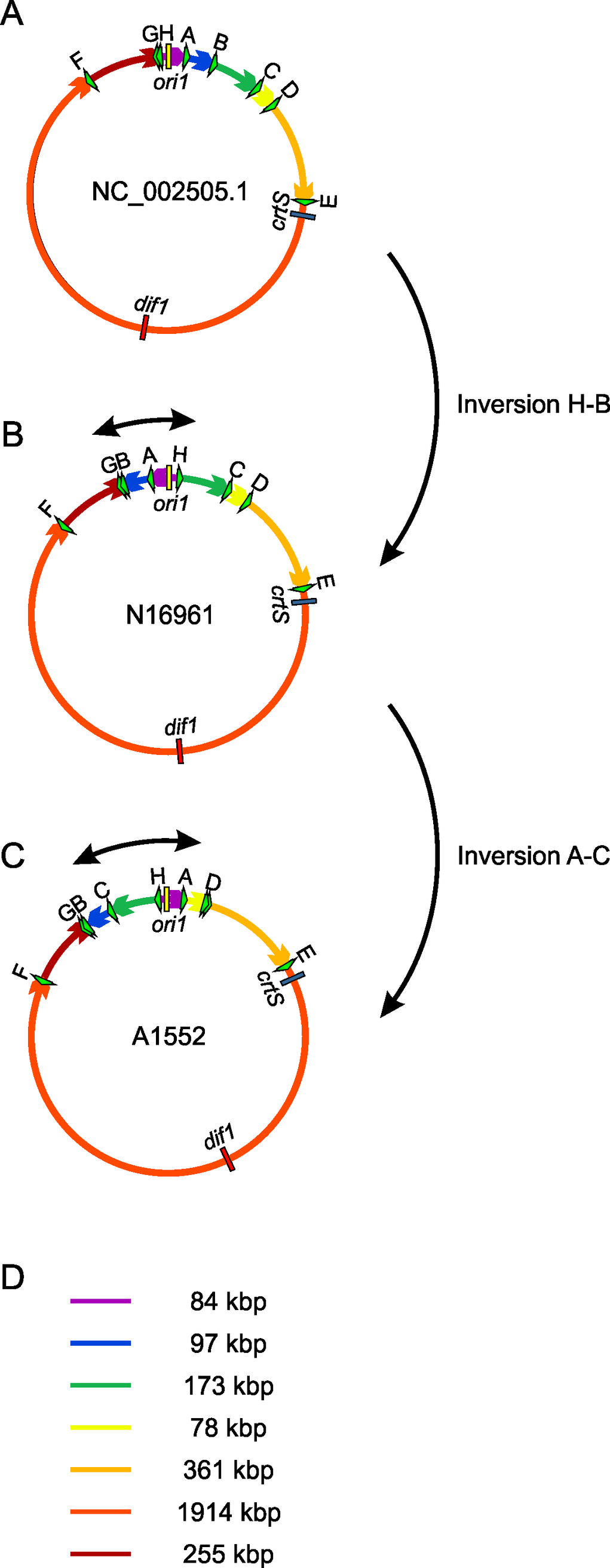

Supplement: S1 Fig — Circles represent Chr1, with green arrows indicating position and orientation of rRNA operons, and rectangles the position of ori1, crtS and difI. Colored lines show the distances between the rRNA operons, arrowheads are for orientation between the maps. Black arrows highlight the inversions. (A) Map of V. cholerae O1 El Tor N16961 Chr1 as described [8]. (B) Map of V. cholerae O1 El Tor N16961 Chr1 as described [25]. (C) Map of V. cholerae A1552 Chr1 as described here. (D) Size of the distances between the rRNA operons, based on NC_002505.1. (TIF) [file pgen.1007251.s001.tif]

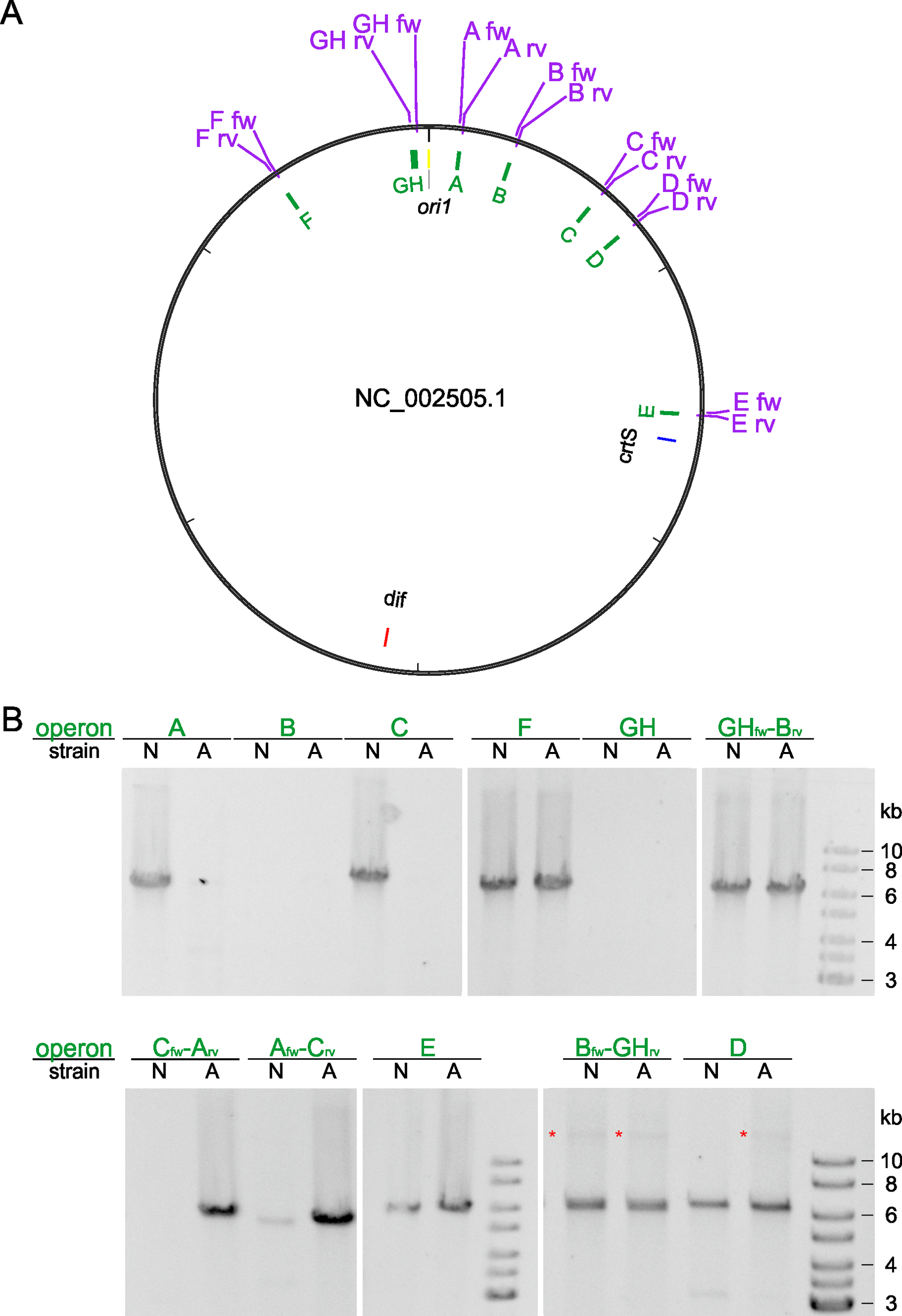

Supplement: S2 Fig — (A) Map of V. cholerae N16961 chrI. Green lines represent rRNA operons, yellow, blue and red lines ori1, crtS and difI, respectively. Purple lines show the binding position of the used primers for diagnostic PCR on the forward (outer ring) and reverse strand (inner ring). Primer sequences are provided in supporting S7 Table. (B) Agarose gels of colony PCR on V. cholerae N16961 (strain “N”) and V. cholerae A1552 (strain “A”). Tested operons and primer combinations are indicated by green letters. All PCRs should give a product of approximately 6 kb, except GH, Bfw-GHrv and D in A1552, which should yield a product of 12 kb (see red asterisks). False 6-kb products in these cases are probably due to sequence similarity in the neighboring rRNA operons. (TIF) [file pgen.1007251.s002.tif]

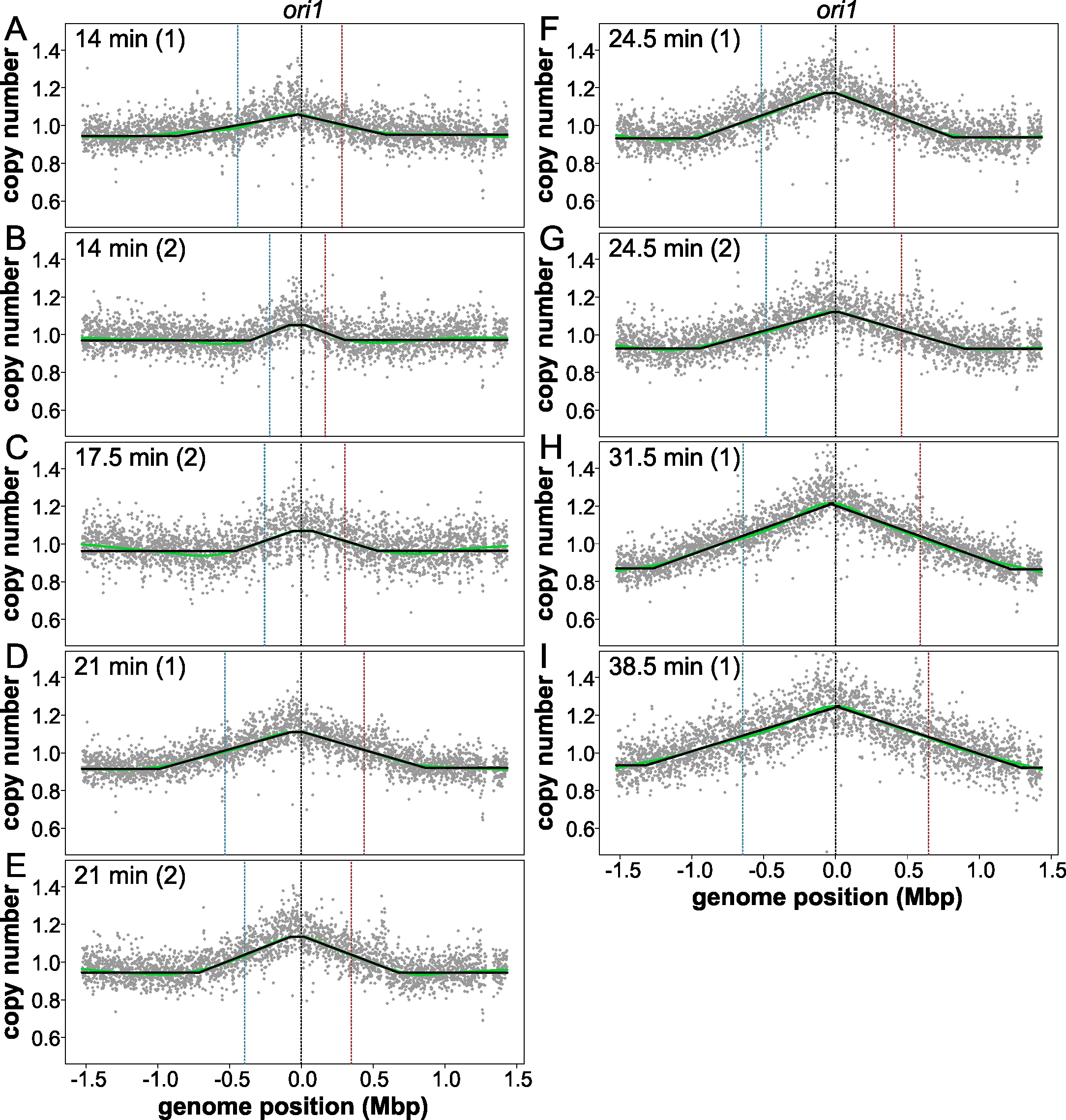

Supplement: S3 Fig — (A-I) Profiles of additional replicates and time points of genome-wide copy numbers after release from stringent response. Derived data are summarized in Fig 2E. (TIF) [file pgen.1007251.s003.tif]

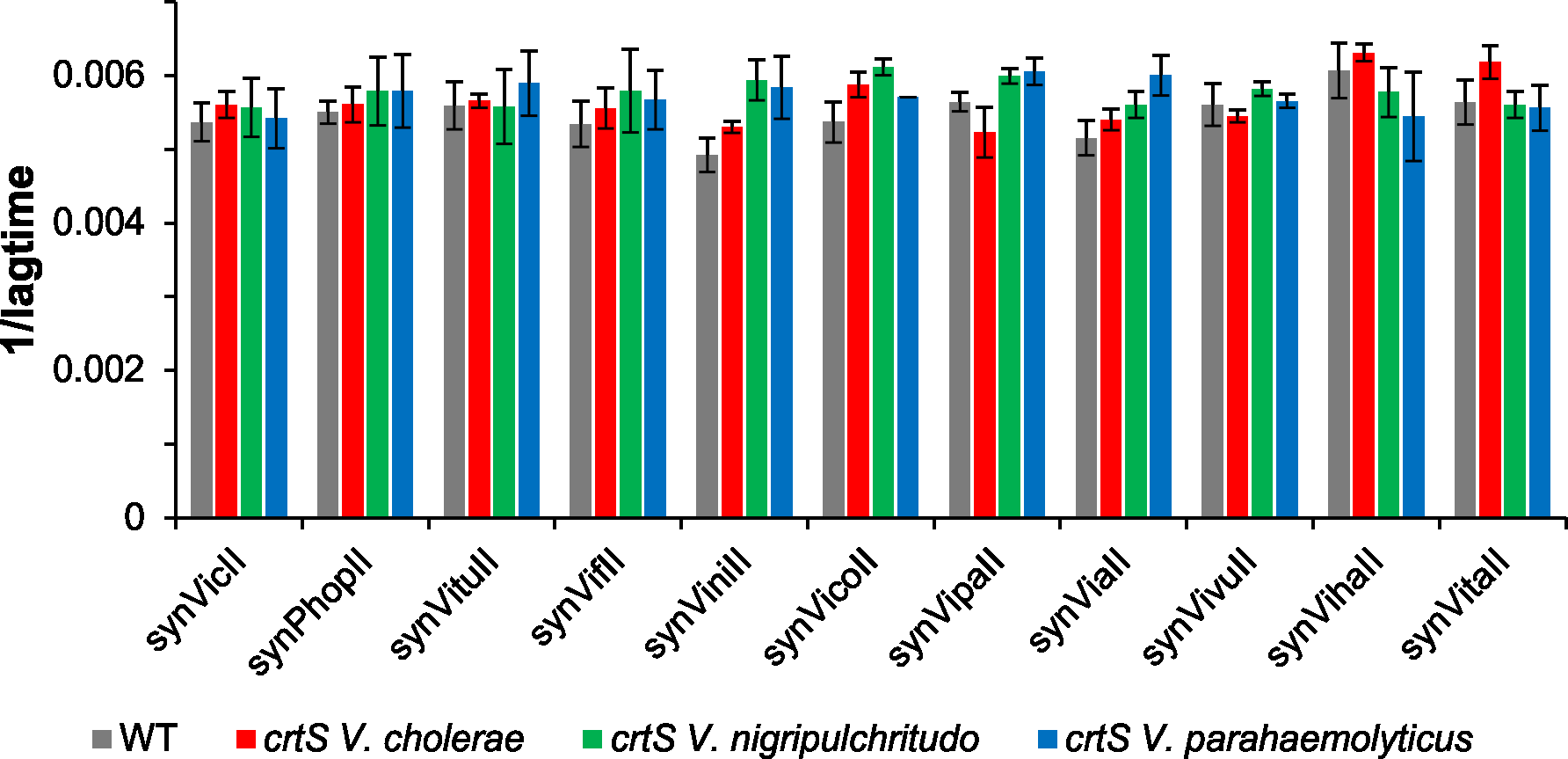

Supplement: S4 Fig — Strains were grown in LB medium with 100 μg/ml ampicillin in a 96-well plate at 37°C. Annotation is as in Fig 5. (TIF) [file pgen.1007251.s004.tif]

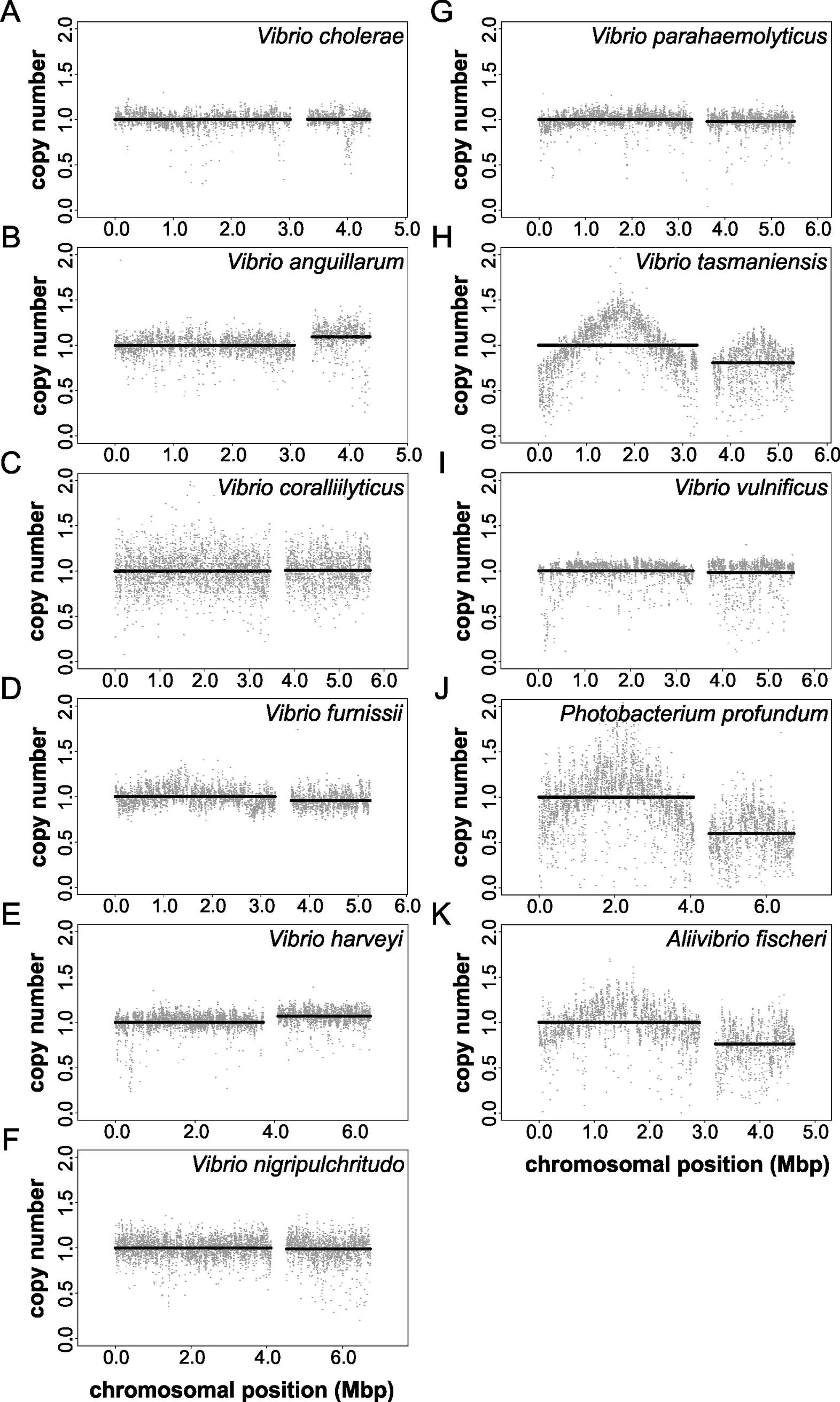

Supplement: S5 Fig — (A-K) Profiles of genome wide copy numbers based on Illumina sequencing. Grey dots represent numbers of reads (normalized to a mean Chr1 copy number of 1). Black lines indicate the mean copy number of each chromosome. (TIF) [file pgen.1007251.s005.tif]

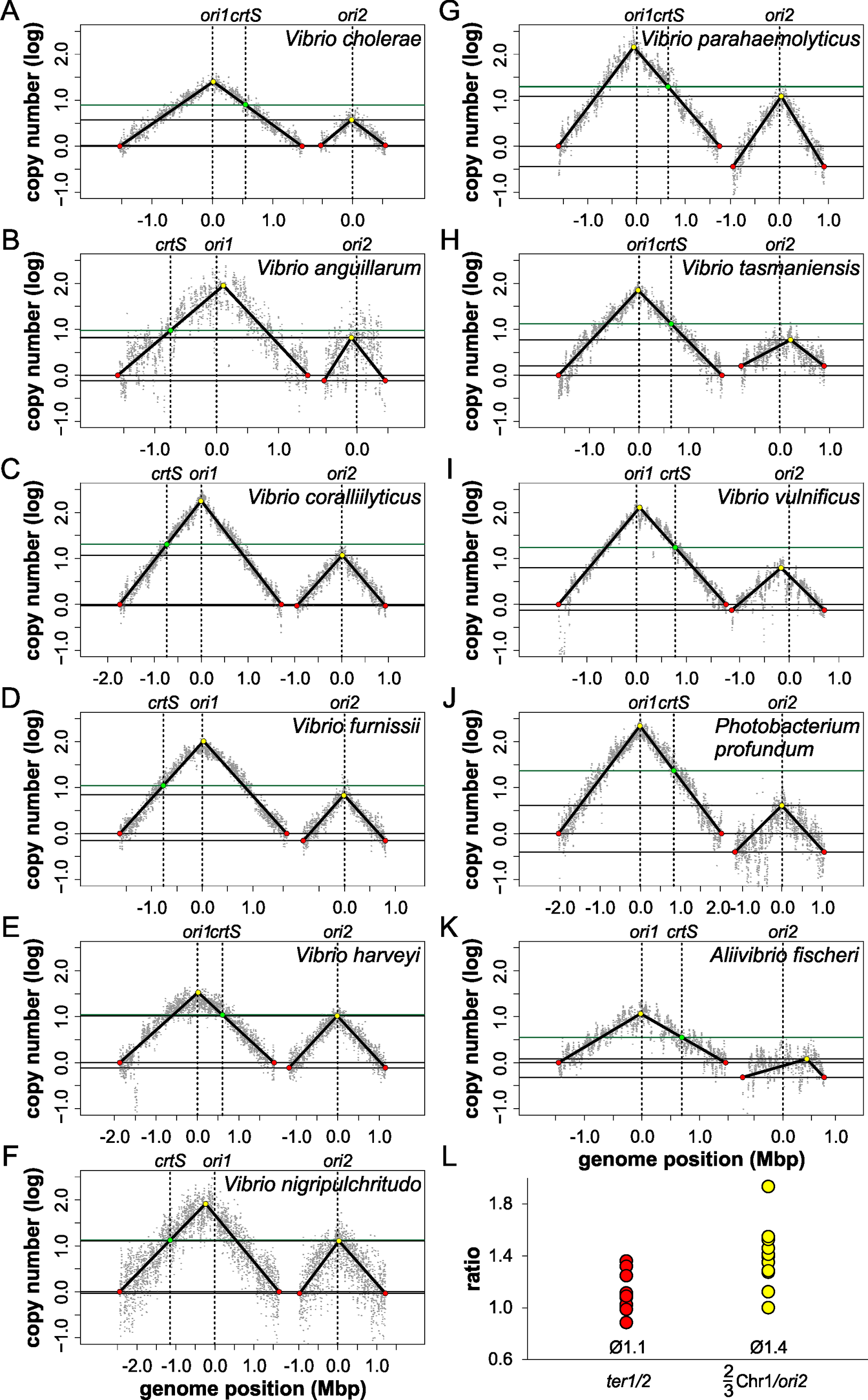

Supplement: S6 Fig — (A-K) Profile of biological replicates of genome wide copy numbers shown in Fig 7 (similar annotation). (TIF) [file pgen.1007251.s006.tif]

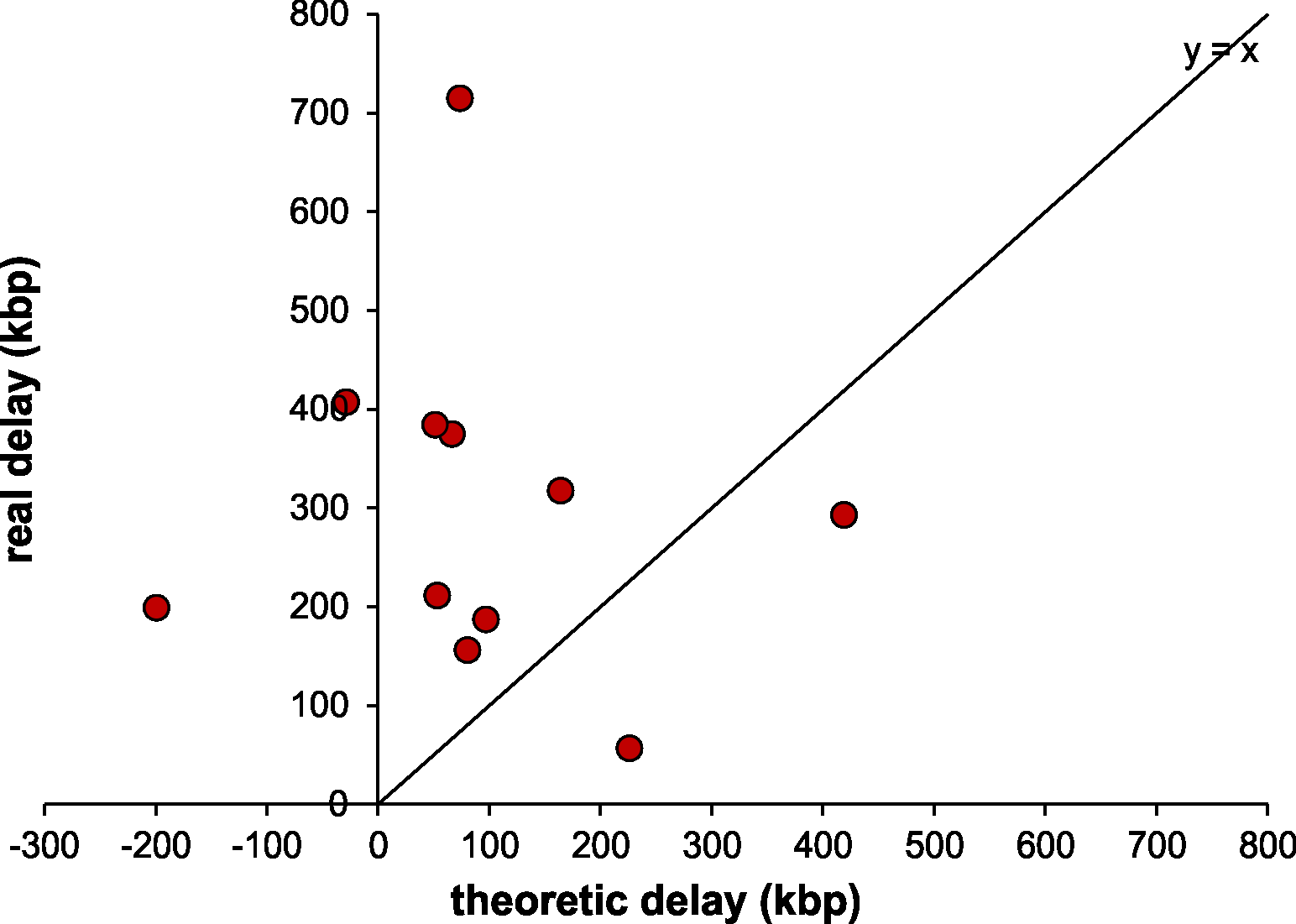

Supplement: S7 Fig — The theoretic delay plotted against the real delay. Theoretic delay is the difference of one half of Chr2 and the distance between crtS and ter1. The real delay is the distance of crtS and the position on Chr1 with the same copy number as ori2 according to the MFA data. Red dots are values from all analyzed strains in Fig 7. The black line has a slope of 1 and simulates perfect correlation. (TIF) [file pgen.1007251.s007.tif]
