# Supplementary material for: Synchronous termination of replication of the two chromosomes is an evolutionary selected feature in Vibrionaceae
Source: PLoS Genet. 2018 Mar 5;14(3):e1007251. doi: 10.1371/journal.pgen.1007251 (PMC5854411; doi:10.1371/journal.pgen.1007251)
Supplement: S1 Table — (PDF) [file pgen.1007251.s008.pdf]

S1 Table. Strains used in comparative genomics

strain	accession number Chr1	accession number Chr2	size Chr1 (bp)	position *ori1*	position *crtS*	size Chr2 (bp)
Vibrio anguillarum 775	NC_015633.1	NC_015637.1	3063912	226620	2478631	988135
Vibrio coralliilyticus OCN014	NZ_CP009264.1	NZ_CP009265.1	3463115	3270180	2536345	1888898
Vibrio furnissii NCTC 11218	NC_016602.1	NC_016628.1	3294546	342209	2880701	1621862
Vibrio harveyi ATCC 43516	NZ_CP014038.1	NZ_CP014039.1	3718332	2026800	2628542	2320549
Vibrio nigripulchritudo SFn1	NC_022528.1	NC_022543.1	4109740	4089489	2941692	2212415
Vibrio parahaemolyticus RIMD 2210633	NC_004603.1	NC_004605.1	3288558	133	639268	1877212
Vibrio cholerae O1 biovar El Tor str. N19961	NC_002505.1	NC_002506.1	2961149	66	817995	1072315
Vibrio cholerae A1552	CP024867	CP024868	3015093	3007817	545922	1070367
Vibrio tasmaniensis LGP32	NC_011753.2	NC_011744.2	3299303	126	647799	1675515
Vibrio vulnificus YJ016	NC_005139.1	NC_005140.1	3354505	118	777384	1857073
Photobacterium profundum SS9	NC_006370.1	NC_006371.1	4085304	4085232	850051	2237943
Vibrio fischeri MJ11	NC_011184.1	NC_011186.1	2905029	101	692029	1418848
Aliivibrio salmonicida LFI1238	NC_011312.1	NC_011313.1	3325165	101	819504	1206461
Vibrio antiquarius /*Vibrio* sp. Ex25	NC_013456.1	NC_013457.1	3259580	427440	3036664	1829445
Vibrio tubiashii ATCC 19109	NZ_CP009354.1	NZ_CP009355.1	3294490	3286785	744726	1766582
Grimontia hollisae strain ATCC 33564	NZ_CP014056.1	NZ_CP014055.1	3215193	1264529	530704	783080
Vibrio mimicus strain ATCC 33654	NZ_CP014043.1	NZ_CP014042.1	3091041	725624	1453717	1347648
Vibrio campbellii (=*Vibrio harveyi* ATCC BAA-1116)	NC_009783.1	NC_009784.1	3765351	416233	1081535	2204018
Vibrio fluvialis strain ATCC 33809	NZ_CP014035.1	NZ_CP014034.1	3155834	2732025	2042979	1671890
Vibrio alginolyticus NBRC 15630 = ATCC 17749	NC_022349.1	NC_022359.1	3334467	2359344	2995211	1812170
Vibrio breoganii strain FF50	NZ_CP016177.1	NZ_CP016178.1	2807405	1405	2224668	1343833
Photobacterium gaetbulicola Gung47	NZ_CP005974.1	NZ_CP005973.1	3856884	325525	3433973	2052529
Vibrio natriegens NBRC 15636 = ATCC 14048 = DSM 759	NZ_CP016345.1	NZ_CP016346.1	3241530	3233842	632484	1927156
Vibrio scophthalmi strain VS-12	NZ_CP016307.1	NZ_CP016308.1	3266565	211644	2680180	1463510
Vibrio tritonius DNA	NZ_AP014635.1	NZ_AP014636.1	3434724	7411	2757564	1787202
Aliivibrio wodanis AWOD1	NZ_LN554846.1	NZ_LN554847.1	3003353	3003243	730195	1515310
Photobacterium damselae subsp. *damselae* strain KC-Na-1	NZ_CP021151.1	NZ_CP021152.1	3134662	3017587	2077199	1105401
Vibrio gazogenes strain ATCC 43942	NZ_CP018835.1	NZ_CP018836.1	3471064	1946502	2759693	1303572
Vibrio owensii strain XSBZ03	NZ_CP019959.1	NZ_CP019960.1	3567557	1839197	1198467	2322648
Vibrio sp. EJY3	NC_016613.1	NC_016614.1	3478307	942	634236	1974339
