# Supplementary material for: Synchronous termination of replication of the two chromosomes is an evolutionary selected feature in Vibrionaceae
Source: PLoS Genet. 2018 Mar 5;14(3):e1007251. doi: 10.1371/journal.pgen.1007251 (PMC5854411; doi:10.1371/journal.pgen.1007251)
Supplement: S2 Table — (PDF) [file pgen.1007251.s009.pdf]

S2 Table. MFA data of figure 7

strain	*Vibrio anguillarum*	*Vibrio corallilyticus*	*Vibrio furnissii*	*Vibrio harveyi*	*Vibrio nigripulchritudo*	*Vibrio parahaemolyticus*	*Vibrio cholerae A1552*	*Vibrio tasmaniensis*	*Vibrio vulnificus*	*Photobacterium profundum*	*Aliivibrio fischeri*
size Chr1 (bp)	3063912	3463115	3294546	3718332	4109740	3288558	3015093	3299303	3354505	4085304	2905029
position *ori1* *	226620	3270180	342209	2026800	4089489	133	3007817	126	118	4085232	101
position max. Chr1	231351	3269658	324898	2014248	3817770	3271966	4223	10558	71663	151	2881434
max. Chr1 – *ori1*	4731	-522	-17311	-12552	-271719	-16725	11499	10432	71545	223	-23696
copy number (log) max. Chr1	1.36	2.29	2.12	1.80	1.96	1.94	1.51	2.04	2.03	1.95	1.09
"position *ter1*"	1758576	1538623	1989482	167634	2034619	1644412	1500271	1649778	1677371	2042580	1452616
position min. Chr1	1741126	1528137	2010439	175794	1667219	1698851	1473372	1675860	1767226	2030604	1467810
copy number (log) min. Chr1	0	0	0	0	0	0	0	0	0	0	0
position *crtS*	2478631	2536345	2880701	2628542	2941692	639268	545922	647799	777384	850051	692029
copy number (log) *crtS*	0.66	1.33	1.12	1.21	1.16	1.20	0.96	1.26	1.19	1.15	0.57
size Chr2 (bp)	988135	1888898	1621862	2320549	2212415	1877212	1070367	1675515	1857073	2237943	1418848
position *ori2* *	534719	1639401	1030429	491600	2733	489	1069748	1675293	1856560	2237753	527
position max. Chr2	514307	1661727	1042245	467249	33179	941	8396	1651187	31830	2211077	1414340
max. Chr2 – *ori2*	-20412	22326	11816	-24351	30446	452	9015	-24106	32343	-26676	5035
copy number (log) max. Chr2	0.61	1.05	0.91	1.03	0.98	0.77	0.65	0.81	0.70	0.46	0.29
"position *ter2*"	947484	1193946	1402364	1651875	1108941	939095	535803	837980	929050	1119162	709951
position min. Chr2	25002	668208	278090	1643769	1084024	913968	597975	927364	882767	1048739	824164
copy number (log) min. Chr2	0.07	0.05	-0.04	0.00	-0.02	-0.40	0.05	0.18	-0.17	-0.58	-0.13
