# Supplementary material for: Synchronous termination of replication of the two chromosomes is an evolutionary selected feature in Vibrionaceae
Source: PLoS Genet. 2018 Mar 5;14(3):e1007251. doi: 10.1371/journal.pgen.1007251 (PMC5854411; doi:10.1371/journal.pgen.1007251)
Supplement: S3 Table — (PDF) [file pgen.1007251.s010.pdf]

S3 Table. MFA data for supporting S6 figure

strain	***Vibrio anguillarum***	***Vibrio corallilyticus***	***Vibrio furnissii***	***Vibrio harveyi***	***Vibrio nigripulchritudo***	***Vibrio parahaemolyticus***	***Vibrio cholerae A1552***	***Vibrio tasmaniensis***	***Vibrio vulnificus***	***Photobacterium profundum***	***Aliivibrio fischeri***
size Chr1 (bp)	3063912	3463115	3294546	3718332	4109740	3288558	3015093	3299303	3354505	4085304	2905029
position *ori1**	226620	3270180	342209	2026800	4089489	133	3007817	126	118	4085232	101
position max. Chr1	337487	3270181	364701	2043689	3855970	3227268	15186	3277433	62805	4078033	2882317
max. Chr1 – *ori1*	110867	1	22492	16889	-233519	-61423	22462	-21996	62687	-7199	-22813
copy number (log) max. Chr1	1.95	2.26	2.01	1.53	1.91	2.16	1.40	1.85	2.11	2.34	1.06
"position *ter1*"	1758576	1538623	1989482	167634	2034619	1644412	1500271	1649778	1677371	2042580	1452616
position min. Chr1	1696405	1534237	2005661	155709	1639472	1689389	1482615	1671236	1793141	2037873	1454954
copy number (log) min. Chr1	0	0	0	0	0	0	0	0	0	0	0
position *crtS*	2478631	2536345	2880701	2628542	2941692	639268	545922	647799	777384	850051	692029
copy number (log) *crtS*	0.98	1.31	1.04	1.04	1.12	1.29	0.90	1.12	1.24	1.37	0.55
size Chr2 (bp)	988135	1888898	1621862	2320549	2212415	1877212	1070367	1675515	1857073	2237943	1418848
position *ori2 **	534719	1639401	1030429	491600	2733	489	1069748	1675293	1856560	2237753	527
position max. Chr2	448752	1648577	1021118	480724	38184	37135	1052428	219506	25150	2226876	413641
max. Chr2 – *ori2*	-85967	9176	-9311	-10876	35451	36646	-17320	219728	25663	-10877	413114
copy number (log) max. Chr2	0.82	1.07	0.84	1.02	1.11	1.09	0.57	0.77	0.80	0.61	0.08
"position *ter2*"	947484	1193946	1402364	1651875	1108941	939095	535803	837980	929050	1119162	709951
position min. Chr2	8229	679698	217776	1641298	1235236	911641	542241	896573	890829	1052370	715483
copy number (log) min. Chr2	-0.11	-0.03	-0.16	-0.11	-0.03	-0.44	0.02	0.20	-0.12	-0.40	-0.32
