# Supplementary material for: Synchronous termination of replication of the two chromosomes is an evolutionary selected feature in Vibrionaceae
Source: PLoS Genet. 2018 Mar 5;14(3):e1007251. doi: 10.1371/journal.pgen.1007251 (PMC5854411; doi:10.1371/journal.pgen.1007251)
Supplement: S4 Table — (PDF) [file pgen.1007251.s011.pdf]

S4 Table. MFA data of stationary phase

strain	copy number Chr1	copy number Chr2
Vibrio anguillarum	1.0	1.09
Vibrio coralliilyticus	1.0	1.01
Vibrio furnissii	1.0	0.96
Vibrio harveyi	1.0	1.07
Vibrio nigripulchritudo	1.0	0.99
Vibrio parahaemolyticus	1.0	0.98
Vibrio cholerae A1552	1.0	1.00
Vibrio tasmaniensis	1.0	0.81
Vibrio vulnificus	1.0	0.98
Photobacterium profundum	1.0	0.60
Aliivibrio fischeri	1.0	0.76
