# Supplementary material for: Synchronous termination of replication of the two chromosomes is an evolutionary selected feature in Vibrionaceae
Source: PLoS Genet. 2018 Mar 5;14(3):e1007251. doi: 10.1371/journal.pgen.1007251 (PMC5854411; doi:10.1371/journal.pgen.1007251)
Supplement: S5 Table — (PDF) [file pgen.1007251.s012.pdf]

S5 Table. Strains used in this study

**Strain**	**Characteristics**	**Resistance**	**Reference**
E. coli AB330	*cf*. DY330, *lacZ*+, *gal*+		Alexander Böhm
E. coli DH5α λpir	F- Φ80*lacZ*ΔM15 Δ(*lacZYA-argF*) U169 *recA1 endA1 hsdR17* (rK-, mK+) *phoA supE44 thi-1 gyrA96 relA1* λ+	nalidixic acid	(1)
E. coli MG1655	Wild type		(2)
E. coli TOP10	F- *mcr*A Δ(*mrr-hsdRMS-mcrBC*) Φ80*lac*ZΔM15 Δ*lacX74* *recA1 araD139* Δ(*ara leu*) 7697 *galU galK rpsL* (StrR) *end*A1 *nupG*	streptomycin	Invitrogen
E. coli XL1Blue	*supE*44, *hsdR*17, *recA*1, *endA*1, *gyrA*96*, thi*1, *relA*1, *lac*$^{-}$ [F' *proAB*, *lac*^{q}ZM15, Tn10(tet^{r})]	tetracycline, nalidixic acid	Stratagene, Agilent Technologies, Germany
V. cholerae A1552		rifampicin	(3)
V. cholerae O1 El Tor N16961		streptomycin	(4)
Aliivibrio fischeri			DSMZ Braunschweig No. 507
Photobacterium profundum			DSMZ Braunschweig No. 21095
Vibrio anguillarum			DSMZ Braunschweig No. 21597
Vibrio corallilyticus			DSMZ Braunschweig No. 19607

Vibrio furnissii			DSMZ Braunschweig No. 14383
Vibrio harveyi			DSMZ Braunschweig No. 19623
Vibrio nigripulchritudo			DSMZ Braunschweig No. 21607
Vibrio parahaemolyticus			DSMZ Braunschweig No. 10027
Vibrio tasmaniensis			DSMZ Braunschweig No. 17182
Vibrio vulnificus			DSMZ Braunschweig No. 10143
E. coli MG1655 FSK102	$crtS_{V.\ nigripulchritudo}$		This work
E. coli MG1655 FSK105	$crtS_{V.\ parahaemolyticus}$		This work
E. coli MG1655 NZ139	$crtS_{V.\ cholerae}$		This work
FSK108	FSK102 synVicII	ampicillin	This work
FSK109	FSK102 synPhopII	ampicillin	This work
FSK110	FSK102 synVituII	ampicillin	This work
FSK111	FSK102 synVifII	ampicillin	This work
FSK112	FSK102 synViniII	ampicillin	This work
FSK113	FSK102 synVicoII	ampicillin	This work
FSK114	FSK102 synVipaII	ampicillin	This work
FSK115	FSK102 synViaII	ampicillin	This work
FSK116	FSK102 synVivuII	ampicillin	This work

FSK117	FSK102 synVitaII	ampicillin	This work
FSK118	FSK102 synVihaII	ampicillin	This work
FSK119	FSK105 synVicII	ampicillin	This work
FSK120	FSK105 synPhopII	ampicillin	This work
FSK121	FSK105 synVituII	ampicillin	This work
FSK122	FSK105 synVifII	ampicillin	This work
FSK123	FSK105 synViniII	ampicillin	This work
FSK124	FSK105 synVicoII	ampicillin	This work
FSK125	FSK105 synVipaII	ampicillin	This work
FSK126	FSK105 synViaII	ampicillin	This work
FSK127	FSK105 synVivuII	ampicillin	This work
FSK128	FSK105 synVitaII	ampicillin	This work
FSK129	FSK105 synVihaII	ampicillin	This work
FSK74	NZ139 synPhopII	ampicillin	This work
FSK75	NZ139 synVituII	ampicillin	This work
FSK76	NZ139 synVifII	ampicillin	This work
FSK77	NZ139 synViniII	ampicillin	This work
FSK78	NZ139 synVicoII	ampicillin	This work
FSK79	NZ139 synVipaII	ampicillin	This work
FSK80	NZ139 synViaII	ampicillin	This work
FSK81	NZ139 synVivuII	ampicillin	This work
FSK93	*E. coli* MG1655 synVihaII	ampicillin	This work
FSK94	NZ139 synVihaII	ampicillin	This work
FSK96	*E. coli* MG1655 synVitaII	ampicillin	This work

FSK97	NZ139 synVitaII	ampicillin	This work
NZ140	NZ139 synVicII	ampicillin	This work
NZ72	*E. coli* MG1655 synVicII	ampicillin	This work
SMS101	*E. coli* MG1655 synVituII	ampicillin	(5)
SMS102	*E. coli* MG1655 synVifII	ampicillin	(5)
SMS106	*E. coli* MG1655 synViniII	ampicillin	(5)
SMS107	*E. coli* MG1655 synVicoII	ampicillin	(5)
SMS108	*E. coli* MG1655 synVipaII	ampicillin	(5)
SMS110	*E. coli* MG1655 synVivuII	ampicillin	(5)
SMS121	*E. coli* MG1655 synPhopII	ampicillin	(5)
SMS134	*E. coli* MG1655 synViaII	ampicillin	(5)

1. Miller VL, Mekalanos JJ. A novel suicide vector and its use in construction of insertion mutations: osmoregulation of outer membrane proteins and virulence determinants in *Vibrio cholerae* requires *toxR*. Journal of bacteriology. 1988;170(6):2575-83. Epub 1988/06/01.
2. Blattner FR, Plunkett G, 3rd, Bloch CA, Perna NT, Burland V, Riley M, et al. The complete genome sequence of *Escherichia coli* K-12. Science. 1997;277(5331):1453-62. Epub 1997/09/05.
3. Yildiz FH, Schoolnik GK. Role of rpoS in stress survival and virulence of Vibrio cholerae. Journal of bacteriology. 1998;180(4):773-84. Epub 1998/02/24.
4. Heidelberg JF, Eisen JA, Nelson WC, Clayton RA, Gwinn ML, Dodson RJ, et al. DNA sequence of both chromosomes of the cholera pathogen *Vibrio cholerae*. Nature. 2000;406(6795):477-83. Epub 2000/08/22.
5. Messerschmidt SJ, Schindler D, Zumkeller CM, Kemter FS, Schallopp N, Waldminghaus T. Optimization and Characterization of the Synthetic Secondary Chromosome synVicII in *Escherichia coli*. Frontiers in bioengineering and biotechnology. 2016;4:96. Epub 2017/01/10.
