# Supplementary material for: Synchronous termination of replication of the two chromosomes is an evolutionary selected feature in Vibrionaceae
Source: PLoS Genet. 2018 Mar 5;14(3):e1007251. doi: 10.1371/journal.pgen.1007251 (PMC5854411; doi:10.1371/journal.pgen.1007251)
Supplement: S6 Table — (PDF) [file pgen.1007251.s013.pdf]

S6 Table. Replicons used in this study

Plasmid	Characteristics	Resistance	Reference
pMA157	MoClo Level 1.2 (pMA349) + *crtS*$_{V. cholerae}$ (coord. 817945-818139)	ampicillin	This work
pMA161	MoClo Level 1.2 (pMA349) + *crtS*$_{V. nigripulchritudo}$ (coord. 2941673-2941868)	ampicillin	This work
pMA207	MoClo assembly of 4 parts into pMA327: pMA709, pMA710, pMA431, pMA157 endlinker pICH50900	kanamycin, spectinomycin	This work
pMA431	MoClo Level 1.3 (pMA350) + *FRT-Kan-FRT*	ampicillin, kanamycin	This work
pMA451	MoClo assembly of 4 parts into pMA327: pMA709, pMA710, pMA431, pMA161 endlinker pICH50900	kanamycin, spectinomycin	This work
pMA454	MoClo assembly of 4 parts into pMA327: pMA709, pMA710, pMA431, pMA892 endlinker pICH50900	Kanamycin, spectinomycin	This work
pMA710	MoClo Level 1.4 (pMA351) + *fucR-rlmM* 3´ flank (coord. 2940123-2940457)	ampicillin	This work

pMA892	MoClo Level 1.2 (pMA349) + *crtS*$_{V. parahaemolyticus}$ (coord. 639262-639456)	ampicillin	This work
synPhopII	synVicII-1.351 + *ori2*$_{P. profundum}$ (coord. 2234799-2736)	ampicillin	(5)
synViaII	synVicII-1.351 + *ori2*$_{V. anguillarum}$ (coord. 531799-537340)	ampicillin	(5)
synVicII-1.352	synVicII-1.35 + *lacZ*	ampicillin	This work
synVicoII	synVicII-1.351 + *ori2*$_{V. coralyticus}$ (coord. 1636573-1642380)	ampicillin	(5)
synVifII	synVicII-1.351 + *ori2*$_{V. furnissii}$ (coord. 1027513-1033467)	ampicillin	(5)
synVihaII	synVicII-1.351 + *ori2*$_{V. harveyi}$ (coord. 489045-494830)	ampicillin	This work
synViniII	synVicII-1.351 + *ori2*$_{V. nigripulchritudo}$ (coord. 2212141-5725)	ampicillin	(5)
synVipaII	synVicII-1.351 + *ori2*$_{V. parahaemolyticus}$ (coord. 1874837-3408)	ampicillin	(5)
synVitaII	synVicII-1.351 + *ori2*$_{V. tasmaniensis}$ (coord. 1672258-2676)	ampicillin	This work
synVituII	synVicII-1.351 + *ori2*$_{V.tubiashi}$ (coord. 1764492-3576)	ampicillin	(5)

synVivuII	synVicII-1.351 + *ori2*$_{V. vulnificus}$ (coord.1853576-2515)	ampicillin	(5)

Genomic positions are indicated according to the following genome annotations: NC_002506.1 for Chr2 of *V. cholerae,* NC_002505.1 for Chr1 of *V. cholerae*, NC_ 000913.2 for *E. coli* MG1655, NC_022528.1 for *V. nigripulchritudo* Chr1, NC_004603.1 for *V. parahaemolyticus* Chr1, for Chr2 of *P. profundum* NC_00637.1, *V. anguillarum* Chr2 NC_015637.1, *V. furnissii* Chr2 NC_016628.1, *V. parahaemolyticus* Chr2 NC_004605.1; *V. vulnificus* Chr2 NC_005140.1, *V. nigripulchritudo* Chr2 NC_022543.1, *V. tubiashi* Chr2 NZ_CP009355.1, *V. coralliilyticus* Chr2 NZ_CP009265.1, *V.harveyi* Chr2 NZ_CP014039.1 and *V. tasmaniensis* Chr2 NC_011744.2 .
