# Supplementary material for: Synchronous termination of replication of the two chromosomes is an evolutionary selected feature in Vibrionaceae
Source: PLoS Genet. 2018 Mar 5;14(3):e1007251. doi: 10.1371/journal.pgen.1007251 (PMC5854411; doi:10.1371/journal.pgen.1007251)
Supplement: S7 Table — (PDF) [file pgen.1007251.s014.pdf]

S7 Table. Oligonucleotides used in this study

Number	Sequence (5´ » 3´)
1002	TTGGTGAGAATCCAAGTAGGGATAATTGCGGCCGCAGCTGGCACGACAGGTTTGCCG
1004	AACTTCGCAGACCTATATTACCCTGATGCGGCCGCGACTATGCGGCATCAGAGC
1164	GAACTTCCTCGAGCATCTCCGACGGCGCGCCAACACAGTTTATCACAGCCA
1410	GAAAAATAAACAAATAGGGGTTCCGCGGGCGCGCCCATTAATCCGGAAAGATGATC
1429	AAGGTCTCGGGAGCGACGCCCCGGCCTTGCCTG
1430	TTGGTCTCCAGCGAAATTCCGTCCGACGCGCAG
1439	AAGGTCTCGGGAGTGAGTGATCTTTTCACTATATTC
1440	TTGGTCTCCAGCGCTCTTTTAAGCAGGATCCTAAC
1443	AAGGTCTCGGGAGCAATTAGATCTTTTTATTAA
1444	TTGGTCTCCAGCGTTACTTTAAGAAAGATCTTA
1455	AAGGTCTCGGGAGGAAGTTCCTATACTTTCTAGAGAATAGGAACTTCGGAATAGGAACTTCAAGATCCCC
1456	TTGGTCTCCAGCGAGTTCCTATTCCGAAGTTCCTATTCTCTAGAAAGTATAGGAACTTCAGAGCGCTTTTG
1474	AAGGTCTCGGGAGACACCTCACATGCAGATCTTTC
1475	TTGGTCTCCAGCGTTTTTTGATCGAATACAAAATATGGATAAATC
1515	GAACTTCCTCGAGCATCTCCGACGGCGCGCCGACATAAAAAAACCGCCCGAAGGC
1516	CCTGGAGATTTTCAATCGTTG
1517	TTCCAACGGAATTTGGCATG

1557	AAAAATAAACAAATAGGGGTTCCGCGGGCGCGCCGCCATGTTT GATCAAGCATC
1704 A fw	GACTGCAAAGAAGAACGCTC
1705 A rv	ATATCGCGAATGACATGCCG
1706 B fw	CAGGGCAATCCATAACATCG
1707 B rv	TAATCCGCTTGTCCAAGCAC
1708 C fw	GATCGAATGTTTGCCGATGC
1709 C rv	TAAAAGCCGATCGTGTCCTC
1710 D fw	GTGTGGGTTACACGAAGATC
1711 D rv	GCCTCAGCAAGAACAACAAC
1712 E fw	TTTTCGCAAGCCAGTAGCAG
1713 E rv	ACGATATGCGCTTCTACTGC
1714 F fw	CGTGATTATCTTAACCCGCG
1715 F rv	AGTATCGTCCTGAGCTATCC
1716 GH fw	AGTGGAGTACACCAATTGGG
1717 GH rv	CCTTCCTCTCTGATTCAGAG
