# Supplementary material for: Synchronous termination of replication of the two chromosomes is an evolutionary selected feature in Vibrionaceae
Source: PLoS Genet. 2018 Mar 5;14(3):e1007251. doi: 10.1371/journal.pgen.1007251 (PMC5854411; doi:10.1371/journal.pgen.1007251)
Supplement: S1 Text — (PDF) [file pgen.1007251.s015.pdf]

Sequencing of *V. cholerae* strain A1552

The Illumina short read and Pacific Bioscience long read sequencing data were assembled *de novo* into a single contig and supported the inversion found by PCR (Supporting S1 figure). To confirm that the chromosomal inversion did not happen recently, for example during transfer from one lab to the other, we sequenced an additional *V. cholerae* A1552 from the Yildiz lab using MinION nanopore sequencing (the other strain came to the Waldminghaus lab through the Blokesch lab). Long sequencing reads spanning the ribosomal operons confirmed the chromosomal inversions, supporting this genome arrangement to be a characteristic of strain A1552. The genome sequence was submitted to GenBank and the strain to the culture collection DSMZ (Accession numbers: GenBank: CP024867; CP024868; Deutsche Sammlung von Mikroorganismen und Zellkultur / German Collection of Microorganisms and Cell Culture: DSM 106276).
